# Structural diverseness of neurons between brain areas and between cases

**DOI:** 10.1038/s41398-020-01173-x

**Published:** 2021-01-14

**Authors:** Ryuta Mizutani, Rino Saiga, Yoshiro Yamamoto, Masayuki Uesugi, Akihisa Takeuchi, Kentaro Uesugi, Yasuko Terada, Yoshio Suzuki, Vincent De Andrade, Francesco De Carlo, Susumu Takekoshi, Chie Inomoto, Naoya Nakamura, Youta Torii, Itaru Kushima, Shuji Iritani, Norio Ozaki, Kenichi Oshima, Masanari Itokawa, Makoto Arai

**Affiliations:** 1 Department of Applied Biochemistry, Tokai University, Hiratsuka, Kanagawa 259-1292 Japan; 2 Department of Mathematics, Tokai University, Hiratsuka, Kanagawa 259-1292 Japan; 3 Japan Synchrotron Radiation Research Institute (JASRI/SPring-8), Sago, Hyogo, 679-5198 Japan; 4 Photon Factory, High Energy Accelerator Research Organization KEK, Tsukuba, Ibaraki 305-0801 Japan; 5 Advanced Photon Source, Argonne National Laboratory, Lemont, IL 60439 USA; 6 Department of Cell Biology, Tokai University School of Medicine, Isehara, Kanagawa 259-1193 Japan; 7 Department of Pathology, Tokai University School of Medicine, Isehara, Kanagawa 259-1193 Japan; 8 Department of Psychiatry, Nagoya University Graduate School of Medicine, Nagoya, Aichi 466-8550 Japan; 9 Medical Genomics Center, Nagoya University Hospital, Nagoya, Aichi 466-8550 Japan; 10 Tokyo Metropolitan Matsuzawa Hospital, Setagaya, Tokyo, 156-0057 Japan; 11 Tokyo Metropolitan Institute of Medical Science, Setagaya, Tokyo, 156-8506 Japan

**Keywords:** Schizophrenia, Physiology

## Abstract

The cerebral cortex is composed of multiple cortical areas that exert a wide variety of brain functions. Although human brain neurons are genetically and areally mosaic, the three-dimensional structural differences between neurons in different brain areas or between the neurons of different individuals have not been delineated. Here we report a nanometer-scale geometric analysis of brain tissues of the superior temporal gyrus of schizophrenia and control cases. The results of the analysis and a comparison with results for the anterior cingulate cortex indicated that (1) neuron structures are significantly dissimilar between brain areas and that (2) the dissimilarity varies from case to case. The structural diverseness was mainly observed in terms of the neurite curvature that inversely correlates with the diameters of the neurites and spines. The analysis also revealed the geometric differences between the neurons of the schizophrenia and control cases. The schizophrenia cases showed a thin and tortuous neuronal network compared with the controls, suggesting that the neuron structure is associated with the disorder. The area dependency of the neuron structure and its diverseness between individuals should represent the individuality of brain functions.

## Introduction

The human cerebral cortex is composed of multiple cortical areas that exert a wide variety of brain functions, such as cognitive functions by the prefrontal cortex and auditory functions by the temporal lobe. Brodmann divided the human cerebral cortex into 52 cortical areas distinguished by their cytoarchitectures and by taking into account functional localization^1,2^. These structurally and functionally different brain areas supposedly operate as modules^3^ that are organized differently between individuals^4–6^. Such large-scale differences between individuals should originate from microscopic differences in neurons that constitute our brain. Although it has been reported that human brain neurons are not monoclonal but rather genetically and areally mosaic due to somatic mutations during developmental stages^7,8^, the three-dimensional structural differences between neurons in different brain areas or between neurons of different individuals have not been delineated.

We previously reported a three-dimensional structural study of neurons of the anterior cingulate cortex of schizophrenia cases and controls^9^. The geometric analysis of the neurons indicated that neurite curvature significantly differs between individuals and the difference become extraordinary in schizophrenia. It has been reported that the anterior cingulate cortex is responsible for emotional and cognitive functions^10,11^ and is associated with mental disorders, including schizophrenia^12,13^. Therefore, the structural differences of neurons in the anterior cingulate cortex should lead to differences in the microcircuits that are relevant to cognitive functions and mental disorders. A further analysis of neuron structures in other brain areas will reveal how our neuron structures differ between brain areas or between cases.

**Fig. 1:**
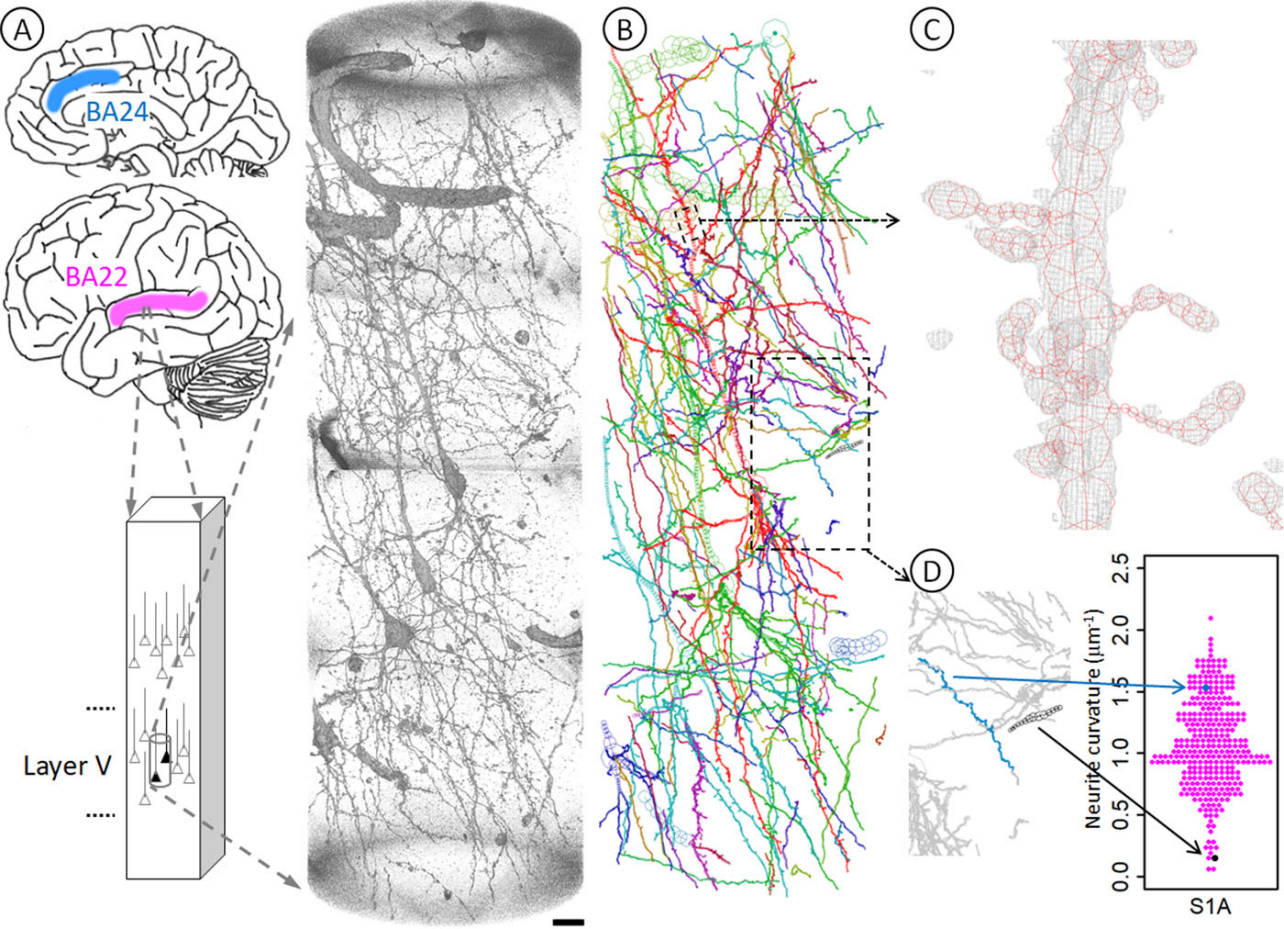
Geometric analysis of neurons at nanometer scale. **A** Brain tissues of Brodmann area 22 (BA22, magenta) of the temporal lobe were stained with Golgi impregnation and subjected to synchrotron radiation nanotomography to visualize three-dimensional tissue structures in layer V. The obtained datasets along with those of the BA24 area (blue) of the anterior cingulate cortex were used for the geometric analysis. The rendering shows a three-dimensional image of the S1A dataset taken from BA22 tissue of the schizophrenia S1 case. The pial surface is toward the top. Voxel values of 160–800 were rendered with the scatter HQ algorithm using VGStudio (Volume Graphics, Germany). Scale bar: 10 μm. **B** The neuronal network was reconstructed in Cartesian coordinate space by tracing structures in the image. First, neurites were scanned by calculating the gradient vector flow^37^ and then traced using a three-dimensional Sobel filter^38^. The resultant computer-generated model was examined and edited as reported previously^9^. The obtained model of the S1A dataset was drawn using MCTrace^22^. Structural constituents are color-coded. The same analysis was repeated for 34 datasets of 4 schizophrenia and 4 control cases. **C** Three-dimensional cage representation of the observed image (gray) is superposed on the structural model of an apical dendrite (red) indicated with a small dashed box in **B**. The three-dimensional map is contoured at 3.0 times the standard deviation (3.0 *σ*) of the voxel values with a grid size of 97.6 nm. Nodes composing the structure are indicated with octagons. **D** Three-dimensional Cartesian coordinates of the traced structure were used to calculate geometric parameters, including average curvature and average torsion for each neurite. A volume indicated with the lower box in **B** is magnified and its two representative neurites are highlighted, of which the blue one is tortuous and hence has a high average curvature, while the black one is rather straight and hence has a low average curvature. Arrows indicate corresponding positions in the beeswarm plot that shows the curvature distribution of all neurites in this S1A structure.

In this study, we analyzed nanometer-scale three-dimensional structures of neurons of Brodmann area 22 (BA22) of the superior temporal gyrus (Fig. 1) of 4 schizophrenia cases (hereafter called S1–S4) and age/gender-matched 4 control cases (N1–N4). The cases used in this study are the same as those of our previous report regarding the neuron structure of the BA24 area of the anterior cingulate cortex^9^. This allowed us to compare neuron structures between the brain areas within each individual. The results of the analysis of the superior temporal gyrus and a comparison with the results for the anterior cingulate cortex revealed structural diverseness of neurons between brain areas and between cases.

## Materials and methods

### Cerebral tissue samples

All post-mortem human cerebral tissues were collected with informed consent from the legal next of kin using protocols approved by the Clinical Study Reviewing Board of Tokai University School of Medicine (application no. 07R-018) and the Ethics Committee of Tokyo Metropolitan Institute of Medical Science (approval no. 17-18). This study was conducted under the approval of the Ethics Committee for the Human Subject Study of Tokai University (approval nos. 11060, 11114, 12114, 13105, 14128, 15129, 16157, 18012, and 19001). The schizophrenia patients S1–S4 and control cases N1–N4 (Supplementary Table 1) of this study were the same with those analyzed in our previous report on the BA24^9^. The number of cases was determined by the available beamtime at the synchrotron radiation facilities. Cerebral tissues of BA22 of the superior temporal gyrus were collected from the left hemispheres of the post-mortem brains and subjected to Golgi impregnation^14^. Our previous results^9,14,15^ indicated that the staining procedure used in this study mainly visualizes neurons and blood vessels. The Golgi-stained tissues were then embedded in borosilicate glass capillaries using epoxy resin, as described previously^9^.

### Microtomography and nanotomography

Overall tissue structures were visualized with simple projection microtomography at the BL20XU beamline^16^ of SPring-8, using monochromatic radiation at 12 keV (Supplementary Table 2). Absorption contrast images of the brain tissues were recorded with a CMOS-based imaging detector (ORCA-Flash4.0, Hamamatsu Photonics, Japan). The layer V position (Supplementary Table 1) of the N3 tissue was estimated from the obtained microtomographic image. The layer V positions of other tissues were estimated from the microtomographic images along with Nissl sections. Examples of the microtomographic images and the Nissl sections are shown in Supplementary Fig. 1. The data collection conditions are summarized in Supplementary Table 2.

Nanotomographic experiments using Fresnel zone plate optics^17^ were performed at the BL37XU beamline^18^ of the SPring-8 synchrotron radiation facility and at the 32-ID beamline^19^ of the Advanced Photon Source (APS) of Argonne National Laboratory, as reported previously^9^. Examples of raw image and reconstructed slice are shown in Supplementary Fig. 2. Photon flux at the sample position was measured using Al_2_O_3_:C dosimeters (Nagase-Landauer, Japan). Spatial resolutions were estimated using three-dimensional test patterns^20^ or from the Fourier domain plot^21^. The experimental conditions are summarized in Supplementary Table 2.

### Structural analysis

Tomographic slices were reconstructed with the convolution-back-projection method using the RecView software, as reported previously^9^. The reconstruction calculation was performed by R.S. The reconstructed datasets were then analyzed with the role allotment of data management to R.S. and data analysis to R.M. R.S. provided the datasets to R.M. in three batches without the case information in order to eliminate human biases in the model building. R.M. analyzed the structure according to the method of our previous study^9^ and returned the number of neurites of each dataset to R.S. without acknowledging the kind of structure represented with that number. No other results were disclosed to R.S. at this point. R.S. aggregated the number according to the case information in order to find cases in which aggregated numbers were <500. The aggregation results were not returned to R.M. Datasets to be further analyzed were chosen by R.S. from predefined candidates and provided to R.M. without the case information.

After R.M. finished building models for all of the datasets, all coordinate files of the structural models in Protein Data Bank format were locked down. Then the case information was disclosed to R.M. Two dummy datasets unrelated to this study were included in order to shuffle datasets taken at the 32-ID beamline. All datasets except these two dummy sets were used in the subsequent analysis. A total of 34 geometric data from the 4 schizophrenia and the 4 control cases were grouped according to the case information and used for the geometric analysis. We found that the data blinding was not essential because the obtained results were unpredictable from the case information.

The three-dimensional Cartesian coordinates of the traced structures were used to calculate geometric parameters, including average curvature and average torsion for each neurite. Figure 1 shows a typical example of the structural analysis. A volume indicated with the lower box in Fig. 1B is magnified and its two representative neurites are highlighted (Fig. 1D), of which the blue one is tortuous and hence has a high average curvature, while the black one is rather straight and hence has a low average curvature. Distribution profiles of these structural parameters were analyzed by using statistical plots, such as beeswarm plots (Fig. 1D).

In these procedures, Cartesian coordinate models were built using the MCTrace software^22^. Geometric parameters were calculated from the obtained structures using the same software. Although user interfaces of MCTrace were updated from the version used in our previous study^9^, we confirmed that the updated and the previous versions gave the same results. The statistics of the obtained structures are summarized in Supplementary Table 3.

### Statistical tests

Statistical tests of structural parameters were performed using the R software for statistical computing^23^. Significance was defined as *p* < 0.05. Differences in structural parameters between cases were examined using Welch’s analysis of variance (ANOVA). Mean neurite curvature and dendritic spine length of the BA22 and BA24 areas of schizophrenia and control cases were analyzed using two-way ANOVA with group (schizophrenia vs. control) and brain area (BA22 vs. BA24) as main factors. The data used for the two-way ANOVA analyses showed no significant difference of the variance in the Bartlett’s test. The equality of the probability distributions was examined using two-sided Kolmogorov–Smirnov tests, and the resultant *p* values were corrected with the Holm–Bonferroni method.

## Results

### Geometric analysis of neurons at nanometer scale

Nanometer-scale three-dimensional structures of the BA22 brain tissues of the schizophrenia S1–S4 and control N1–N4 cases (Supplementary Table 1) were visualized by using synchrotron radiation nanotomography (Supplementary Tables 1–3)^17–19^. A total of 34 three-dimensional image datasets of layer V of the BA22 cortex were blinded by coding dataset names and subjected to a computerized procedure^9^ to build Cartesian coordinate models of tissue structures (Fig. 1, Supplementary Video 1 and Supplementary Figs. 3–5). After the model-building procedures for all datasets were completed, the blinded datasets were re-assigned to the cases and analyzed by calculating geometric parameters including curvature and torsion of neurites, which represent the reciprocal of the radius of three-dimensional neurite curve and the deviation of the curve from a plane, respectively.

**Fig. 2:**
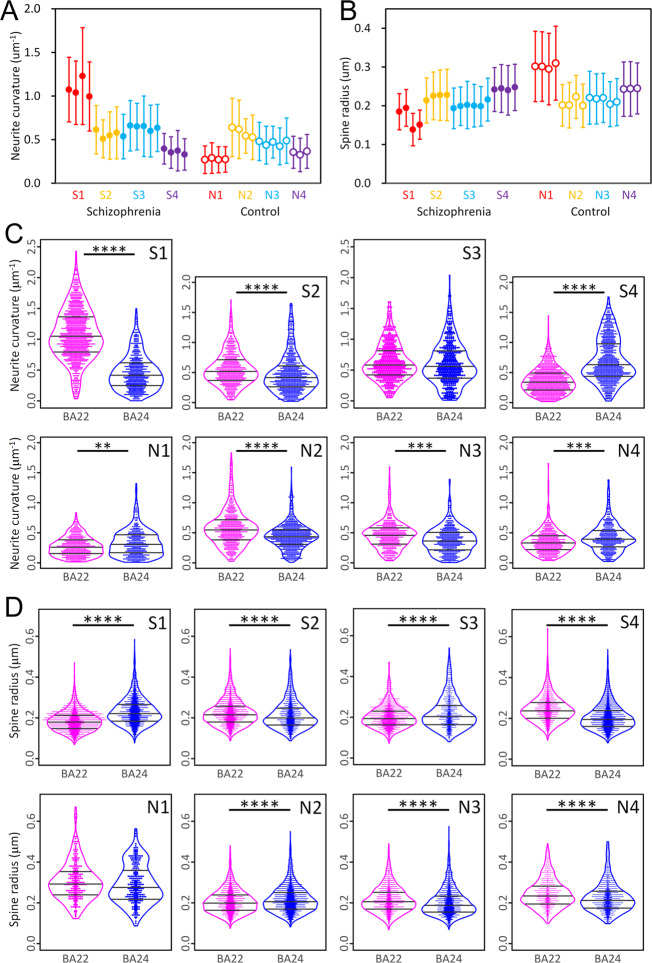

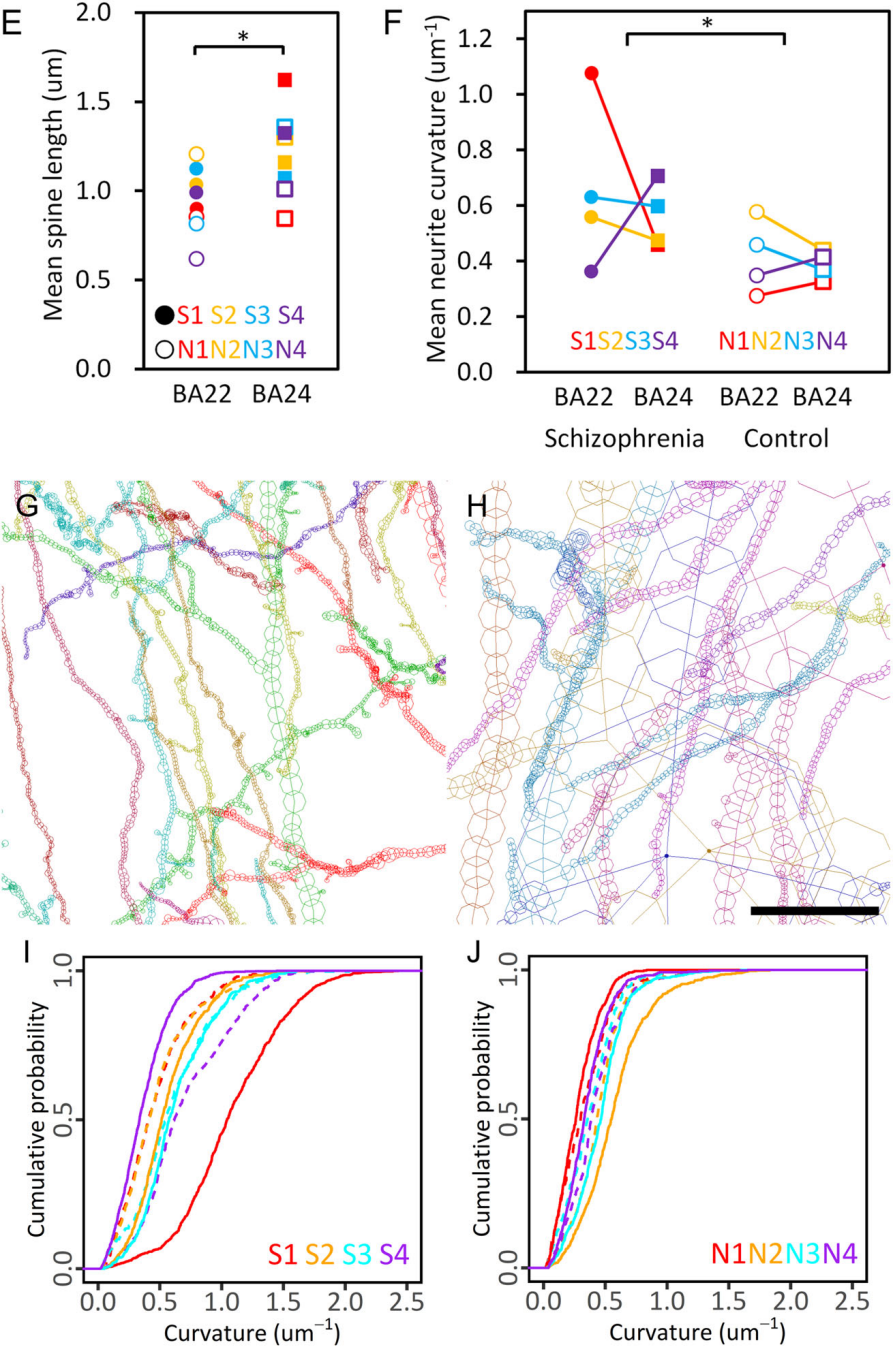
Differences in neuron structure between brain areas and between cases. **A**, **B** Neurite curvature (**A**) and spine thickness radius (**B**) of temporal (BA22) cortex calculated from each dataset vary depending on the cases (*p* = 2.9 × 10^−8^ and *F* = 91.7, for neurite curvature, *p* = 4.5 × 10^−8^ and *F* = 74.9, for spine radius, Welch’s ANOVA with case as the main factor). Schizophrenia cases S1–S4 and age/gender-matched control cases N1–N4 are color-coded. Neurite curvature was calculated by averaging the curvature along the neurite. Spine radius was calculated by averaging the thickness radius along the dendritic spine. Circles indicate mean values of each dataset, and error bars indicate standard deviation. **C**, **D** Frequency distribution of neurite curvature (**C**) and spine radius (**D**) of temporal (BA22) and prefrontal (BA24) cortexes of each case. Schizophrenia cases S1–S4 and controls N1–N4 are indicated with labels. The equality of distributions between BA22 and BA24 was examined using Kolmogorov–Smirnov test, and their *p* values were corrected with the Holm–Bonferroni method. ****p < 10^−8^; ***p < 10^−4^; **p < 10^−2^. Quartiles are indicated with bars. Dot size is adjusted for visibility. Differences in neuron structure between brain areas and between cases. **E** Mean spine length is significantly shorter in BA22 than in BA24 (*p = 0.023, *F* = 6.7, two-way ANOVA with group (schizophrenia/control) and brain area (BA22/BA24) as main factors). Schizophrenia cases S1–S4 and controls N1–N4 are color-coded. **F** Mean neurite curvature is significantly higher in the schizophrenia cases than in the controls (*p = 0.031, *F* = 5.9, two-way ANOVA with group (schizophrenia/control) and brain area (BA22/BA24) as main factors). **G** Neurites of BA22 of the schizophrenia S1 case are tortuous and thin, **H** while those of the age/gender-matched control N1 case are smooth and thick. Structures of S1A and N1A are drawn to the same scale using MCTrace^22^. The pial surface is toward the top. Structural constituents are color-coded. Nodes composing each constituent are indicated with octagons. The color coding and structural orientation are the same as in Fig. 1B and Supplementary Fig. 5B. Scale bar: 10 μm. **I**, **J** Cumulative distribution of neurite curvature of schizophrenia cases (**I**) and controls (**J**). Solid lines represent BA22 distributions, and dashed lines represent BA24 distributions. Cases are color-coded.

The obtained results of the BA22 temporal cortex indicated that the structural parameters calculated from each dataset stepwisely vary depending on the cases (Fig. 2A, B; significance was defined as *p* < 0.05). This suggests that the BA22 of each individual has its own cellular geometry. Next, we compared the structural parameters of the BA22 and BA24 cortexes and found that the frequency distribution of neurite curvature significantly differs between the brain areas for both the schizophrenia and control cases except for the schizophrenia S3 case (Fig. 2C). In contrast, the frequency distribution of neurite torsion showed similar zero-centered profiles for all cases (Supplementary Fig. 6A). The magnitude relation of neurite curvatures of BA22 and BA24 is not consistent and varies from case to case. In the control N2 case for example, the mean neurite curvature of BA22 (0.58 μm^−1^; Supplementary Tables 1 and 4) is 1.31 times higher than that of BA24 (0.44 μm^−1^), while in the control N4 case, the mean neurite curvature of BA22 (0.35 μm^−1^) is 0.84 times lower than that of BA24 (0.41 μm^−1^). The distribution profiles themselves vary depending on the brain area. In the N3 control case for example, the frequency distribution of the neurite curvature of BA22 shows a diamond-shaped profile in contrast to the drop-shaped profile of BA24 (Fig. 2C), indicating that low-curvature neurites are rather minor in BA22 compared to BA24. The frequency distributions of the mean thickness radii and lengths of dendritic spines also indicate significant differences between BA22 and BA24 for both the schizophrenia and control cases, except for the control N1 case (Fig. 2D and Supplementary Fig. 6B). The frequency distribution of the spine length of BA22 shows a bell-shaped profile in every case (Supplementary Fig. 6B), while that of BA24 shows drop-shaped profiles in the S1 and the N3 cases or long upper tails in the S4 and N2 cases. This results in a significant difference in mean spine length between the BA22 and the BA24 areas (Fig. 2E). These structural differences of neurites and spines between brain areas and between cases indicate that (1) neuron structures are dissimilar between the brain areas in every individual and that (2) the dissimilarity itself varies from person to person. The neurite curvature determines the spatial trajectory of neurites and hence affects neuronal microcircuits. The spine radius and length determine the local synaptic potential and hence affect the electrophysiological activity of neurons^24^. Therefore, the area/case dependency of neuron structures can influence the functional performance of their brain areas.

### Geometric differences between schizophrenia and control cases

The geometric analyses also revealed structural differences between schizophrenia cases and controls. The frequency distribution of neurite curvature of the schizophrenia cases shows fat upper tails (Fig. 2C), resulting in a significantly higher mean curvature in the schizophrenia cases than in the controls (Fig. 2F). Consequently, the neurites of the schizophrenia cases are tortuous and thin (Fig. 2G), while the neurites of the control cases are smooth and thick (Fig. 2H). Since the thinning of the neurites and spines can reduce the tissue volume, the volumetric changes in schizophrenia brains^25–27^ should originate from these microscopic structural changes in neurons. The neurite curvature of the schizophrenia cases show larger deviations between cases and between areas compared with the controls (Fig. 2I, J). In the schizophrenia S1 case for example, the mean neurite curvature of BA22 (1.08 μm^−^^1^; Supplementary Table 1) is 2.3 times higher than that of BA24 (0.46 μm^−1^), while in the schizophrenia S4 case, the mean neurite curvature of BA22 (0.36 μm^−1^) is 0.51 times lower than that of BA24 (0.71 μm^−1^). Mean spine radius shows magnitude relations opposite to those of neurite curvature. In the S1 case for example, the spine radius is thinner in BA22 than in BA24, while neurite curvature is higher in BA22 than in BA24 (Fig. 2D).

### Common structural features of neurons

**Fig. 3:**
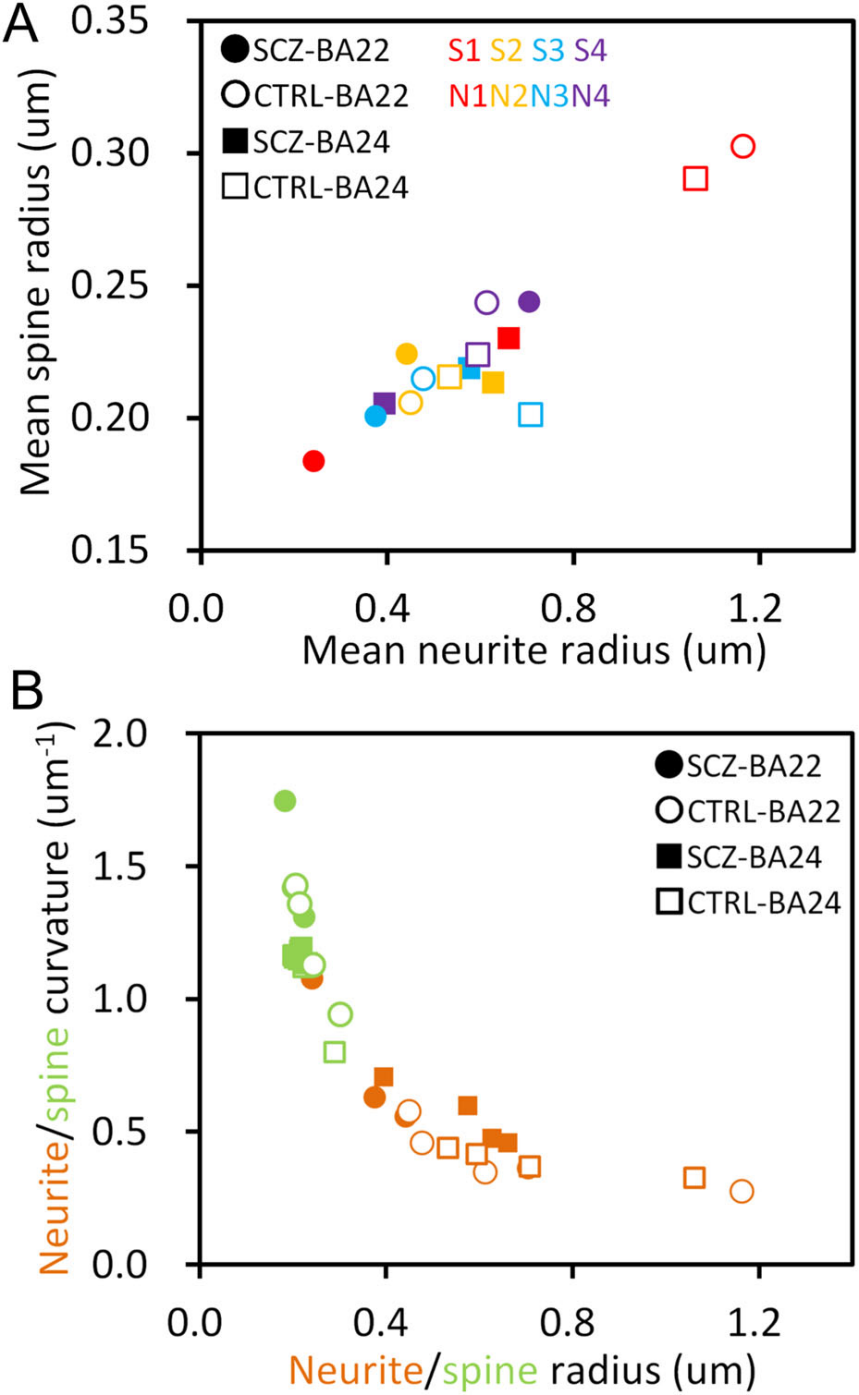
Common structural features of neurons. **A** Linear correlation between mean thickness radii of neurites and spines. Arithmetic mean of neurite radii or spine radii was calculated for BA22 or BA24 of each case. Schizophrenia cases S1–S4 and controls N1–N4 are color-coded. **B** Reciprocal relationship between curvature and thickness radius. Arithmetic mean of curvature or radius of neurites (orange) or spines (green) was calculated for BA22 or BA24 of each case.

Common structural features of neurons across brain areas and across cases were also identified from the structural analysis. Figure 3A shows the relationship between mean thickness radius of neurites and that of spines. The plot shows a linear correlation, indicating that mean spine thickness correlates with mean neurite radius. This suggests that these functionally different elements are structurally constrained in the neuron. Another common feature of the neuron structure was found in the relationship between curvature and thickness. Since a thin thread can sharply curve, whereas a thick thread cannot, curvature is reciprocal to thickness radius as a general rule. A plot of this relationship (Fig. 3B) shows in all the cases that both neurites and spines follow the same reciprocal relationship independently of the brain area. This suggests that both neurites and spines follow a certain common physical or geometric principle that governs their structural constitution.

## Discussion

This study revealed the structural diverseness of neurons between brain areas and between cases. The geometric analysis identified inter-area/case differences of neurite curvature (Fig. 2C, F) that reciprocally correlates with neurite thickness (Fig. 3B). According to the cable theory, neurite diameter affects the propagation distance of the action potential. Indeed, it has been reported that the neurite diameter is related with the action potential amplitude^28^. The linear correlation of neurite thickness to that of spines (Fig. 3A) indicates that neurite thinning narrows spines. Spine size associates with the long-term potentiation^29^ and also with spine substructures, such as postsynaptic density^30,31^. Therefore, thinning of neurites and spines alters the firing efficiency of neurons^24^ and affects the activity of their brain area. The geometric analysis also indicated that these functionally relevant structures differ from area to area and from case to case (Fig. 2). This coincides with the fact that each individual has a unique functional spectrum in their brain and hence has his or her own talents.

The schizophrenia cases showed a thin and tortuous neuronal network compared with the controls (Fig. 2F–H), suggesting that the neuron structure is associated with the disorder. The diverseness of neurite curvature between brain areas was rather large in the schizophrenia cases (Fig. 2I, J). The S1 case showed the highest neurite curvature in BA22 but a rather low curvature in BA24 (Fig. 2F). In contrast, the relationship is reversed in the S4 case: the neurite curvature of BA22 is low, while that of BA24 is high. These results imply that the brain areas compensate each other. However, the large heterogeneity of neurons in the schizophrenia cases can cause functional imbalances between brain areas that may result in disorders of total brain function. The large-scale heterogeneity of schizophrenia brains observed in magnetic resonance imaging (MRI) studies^32,33^ is ascribable to a microscopic heterogeneity of brain tissue. However, we have no consensus regarding the neuropathology of schizophrenia at present^34^. The area-dependent heterogeneous nature of the brain tissue of the schizophrenia cases leads to histological differences between cases and between brain areas and thereby gives rise to seemingly unreproducible results. We suggest that the controversy in the neuropathology of schizophrenia represents the area/case-dependent heterogeneity of neurons in schizophrenia.

The geometric diverseness of neurons found in this study cannot be delineated without conducting three-dimensional analyses of human brain tissues. An advantage of three-dimensional tissue analyses is that the structural difference can be quantitatively examined. Although neuron structures have been visualized using histological sections, structures along the viewing direction, such as spines behind neurites, are missed in light microscopy images, resulting in methodological biases in the subsequent analysis. In contrast, three-dimensional analyses allow us to examine exact structures by using mathematically defined parameters. A major limitation of this study is that the number of analyzed cases was limited by the availability of synchrotron radiation beamtime for the three-dimensional visualization. Although thousands of neurites and tens of thousands of spines were analyzed from their three-dimensional coordinates, the present results are based on brain tissues of just 4 schizophrenia and 4 control cases. The analysis of two Brodmann areas of these cases took nearly 10 years of synchrotron radiation experiments and subsequent structural analyses, but this should be repeated for additional cases in order to further examine the geometric diverseness of neurons. Another limitation of our analysis is the staining property of the Golgi method used for visualizing neurons in the X-ray image. Although the Golgi method has long been used for morphological studies of neurons, it does not stain every neuron and only visualizes a subset of neurons. Therefore, the analyzed neurons should be regarded as representative examples of the entire neuron population. It is also possible that antipsychotics caused the inter-area/case heterogeneity in the schizophrenia cases, though such an area-dependent heterogeneous action of therapeutics can be ascribed to (1) heterogeneous distribution of the therapeutics and/or (2) heterogeneous reaction of neurons to the therapeutics. Since these pharmacokinetic or physiological heterogeneities should be associated with the genetic mosaicism of human brain neurons^7,8^, the relationship between the action of therapeutics and the genetic mosaicism should be further investigated in a number of schizophrenia cases.

Our nanometer-scale three-dimensional study of human brain tissues revealed the structural diverseness of neurons between brain areas and between cases. The brain has a modular architecture^35,36^ in which different areas with different genetic backgrounds^7,8^ exert different functions^2^. Besides the genetic mosaicism, environmental factors and education differences between individuals provide different stimuli to different brain areas, possibly resulting in differences in neuron structures between brain areas. The neurite curvature of the S4 case carrying a frameshift mutation in the *GLO1* gene was highest for BA24^9^ but lowest for BA22 among the schizophrenia cases (Fig. 2F). These results indicate that the neurite curvature is not ascribable solely to a single mutation. Rather, the structural diverseness between brain areas should have originated from multiple factors that affected neuron structures throughout the life of each individual. We suggest that further studies on neuron geometry and its relation with person-associated factors will shed light on the physical basis of the individuality of our brains.

## Supplementary information

Supplementary Video 1

Suppl Figures 1-6, Table 4, Video caption

Supplementary Tables 1-3

## Data Availability

RecView software^15^ and its source code used for the tomographic reconstruction are available from https://mizutanilab.github.io under the BSD 2-Clause License. The model building and geometric analysis procedures were implemented in the MCTrace software available from the same site under the BSD 2-Clause License.
