# Supplementary material for: Structural diverseness of neurons between brain areas and between cases
Source: Transl Psychiatry. 2021 Jan 14;11:49. doi: 10.1038/s41398-020-01173-x (PMC7809156; doi:10.1038/s41398-020-01173-x)
Supplement: Supplementary file 2 — Suppl Figures 1-6, Table 4, Video caption [file 41398_2020_1173_MOESM2_ESM.pdf]

Supplementary Materials of:

Structural diverseness of neurons between brain areas and between cases

Ryuta Mizutani PhD[1]*, Rino Saiga BS[1], Yoshiro Yamamoto PhD[2], Masayuki Uesugi PhD[3], Akihisa Takeuchi PhD[3], Kentaro Uesugi PhD[3], Yasuko Terada PhD[3], Yoshio Suzuki PhD[4], Vincent De Andrade PhD[5], Francesco De Carlo PhD[5], Susumu Takekoshi PhD[6], Chie Inomoto PhD MD[7], Naoya Nakamura PhD MD[7], Youta Torii PhD MD[8], Itaru Kushima PhD MD[8,9], Shuji Iritani PhD MD[8,10], Norio Ozaki PhD MD[8], Kenichi Oshima PhD MD[10,11], Masanari Itokawa PhD MD[10,11], and Makoto Arai PhD[11]

[1]Department of Applied Biochemistry, Tokai University, Hiratsuka, Kanagawa 259-1292, Japan; [2]Department of Mathematics, Tokai University, Hiratsuka, Kanagawa 259-1292, Japan; [3]Japan Synchrotron Radiation Research Institute (JASRI/SPring-8), Sayo, Hyogo 679-5198, Japan; [4]Photon Factory, High Energy Accelerator Research Organization KEK, Tsukuba, Ibaraki 305-0801, Japan; [5]Advanced Photon Source, Argonne National Laboratory, Lemont, IL 60439, USA; [6]Department of Cell Biology, Tokai University School of Medicine, Isehara, Kanagawa 259-1193, Japan; [7]Department of Pathology, Tokai University School of Medicine, Isehara, Kanagawa 259-1193, Japan; [8]Department of Psychiatry, Nagoya University Graduate School of Medicine, Nagoya, Aichi 466-8550, Japan; [9]Medical Genomics Center, Nagoya University Hospital, Nagoya 466-8550, Aichi, Japan; [10]Tokyo Metropolitan Matsuzawa Hospital, Setagaya, Tokyo 156-0057, Japan; [11]Tokyo Metropolitan Institute of Medical Science, Setagaya, Tokyo 156-8506, Japan
*mizutanilaboratory@gmail.com

Index

Supplementary Figure 1 ... p. 2
Supplementary Figure 2 ... p. 3
Supplementary Figure 3 ... p. 4-10
Supplementary Figure 4 ... p. 11-17
Supplementary Figure 5 ... p. 18-23
Supplementary Figure 6 ... p. 24
Supplementary Table 4 ... p. 25
Supplementary Video caption ... p. 26

Supplementary Tables 1-3 are provided separately.

A **B** **C** **D**

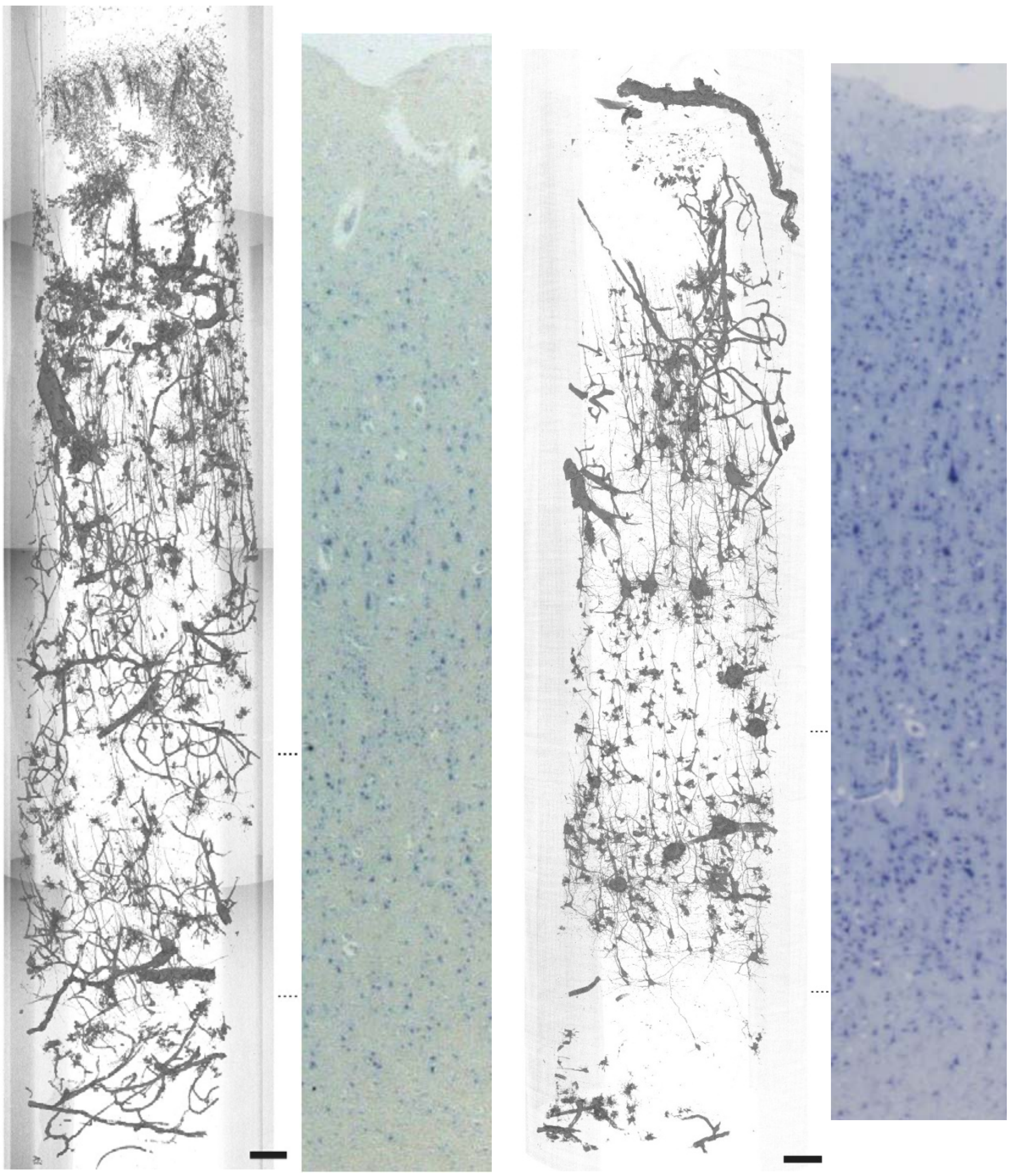

Supplementary Figure 1. Tissue images of S2 and N2 samples. Dotted lines indicate layer V positions. Nissl sections were scaled so as to match the microtomographic images. Scale bars: 100 μm. (**A**) Three-dimensional structure of the S2 sample visualized with microtomography. Linear attenuation coefficients of 16–56 cm^{-1} were rendered with the scatter HQ algorithm using the VG Studio software. (**B**) Nissl section of S2. Contrast was adjusted for visibility. (**C**) Three-dimensional structure of the N2 sample. Linear attenuation coefficients of 16–56 cm^{-1} were rendered. (**D**) Nissl section of N2.

A

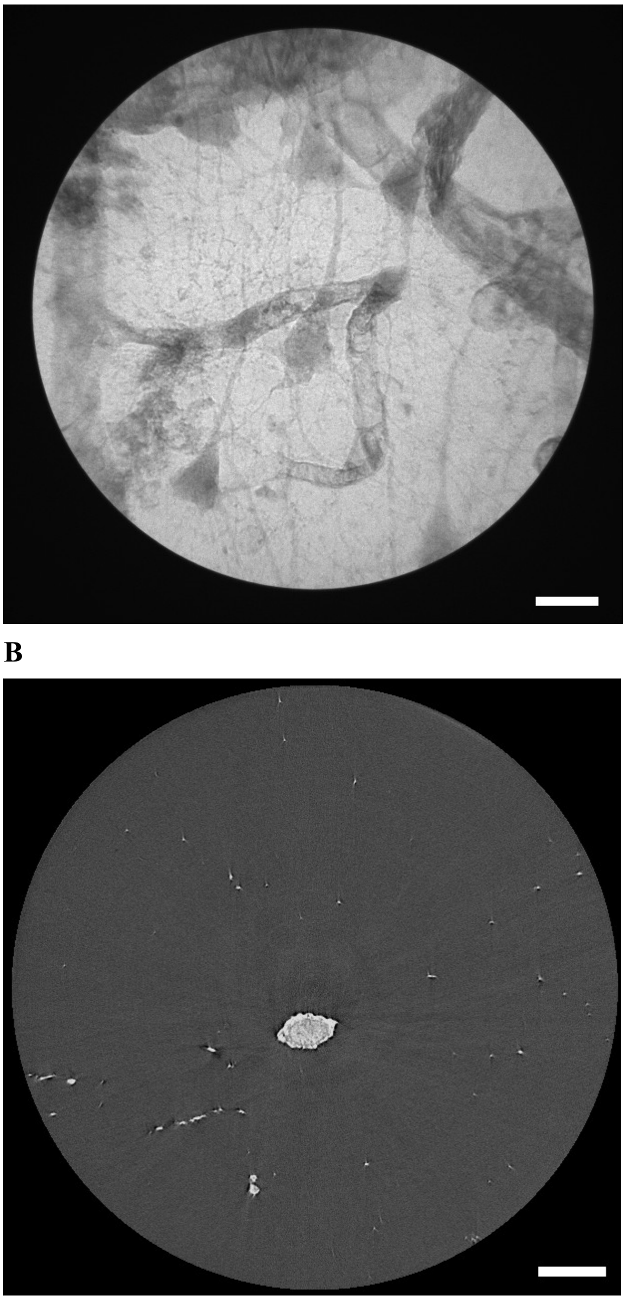

Supplementary Figure 2. Absorption-contrast raw image (**A**) and reconstructed slice (**B**) of dataset S1A of the schizophrenia S1 case. A three-dimensional rendering of this structure is shown in Fig 1a. Scale bars: 10 µm. (**A**) The pyramidal neuron at the center of the image corresponds to the red pyramidal neuron seen in Fig 1b. Pixel values of 0 to 5000 were linearly gray-scaled. (**B**) Tomographic slice at the soma of the center neuron of panel **A**. Voxel values of -400 to 1000 were linearly gray-scaled.

A **B**

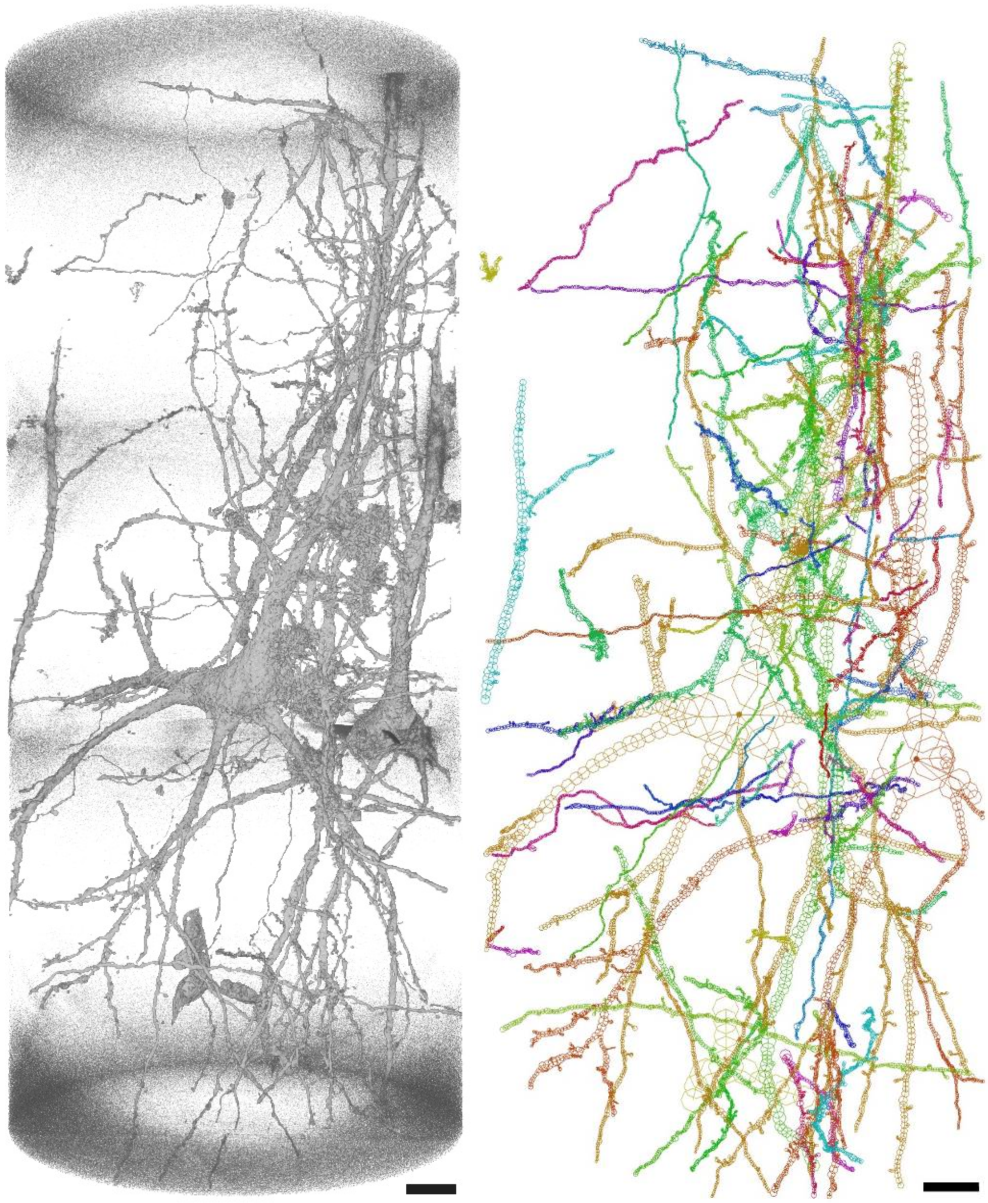

Supplementary Figure 3. Rendering of three-dimensional images of cerebral tissues and Cartesian coordinate models of the tissue structures. Renderings and models are viewed from nearly the same direction. The pial surface is toward the top. Images were rendered with the scatter HQ algorithm of the VG Studio software. Voxel values of 160–800 were rendered in gray scale. Models were drawn with the MCTrace software. Model constituents are color-coded. Nodes composing each constituent are indicated with octagons. Dots indicate somata nodes. Scale bars: 10 μm. (**A**) Rendering of image dataset S2B of the schizophrenia S2 case. (**B**) Cartesian coordinate model of S2B.

C **D**

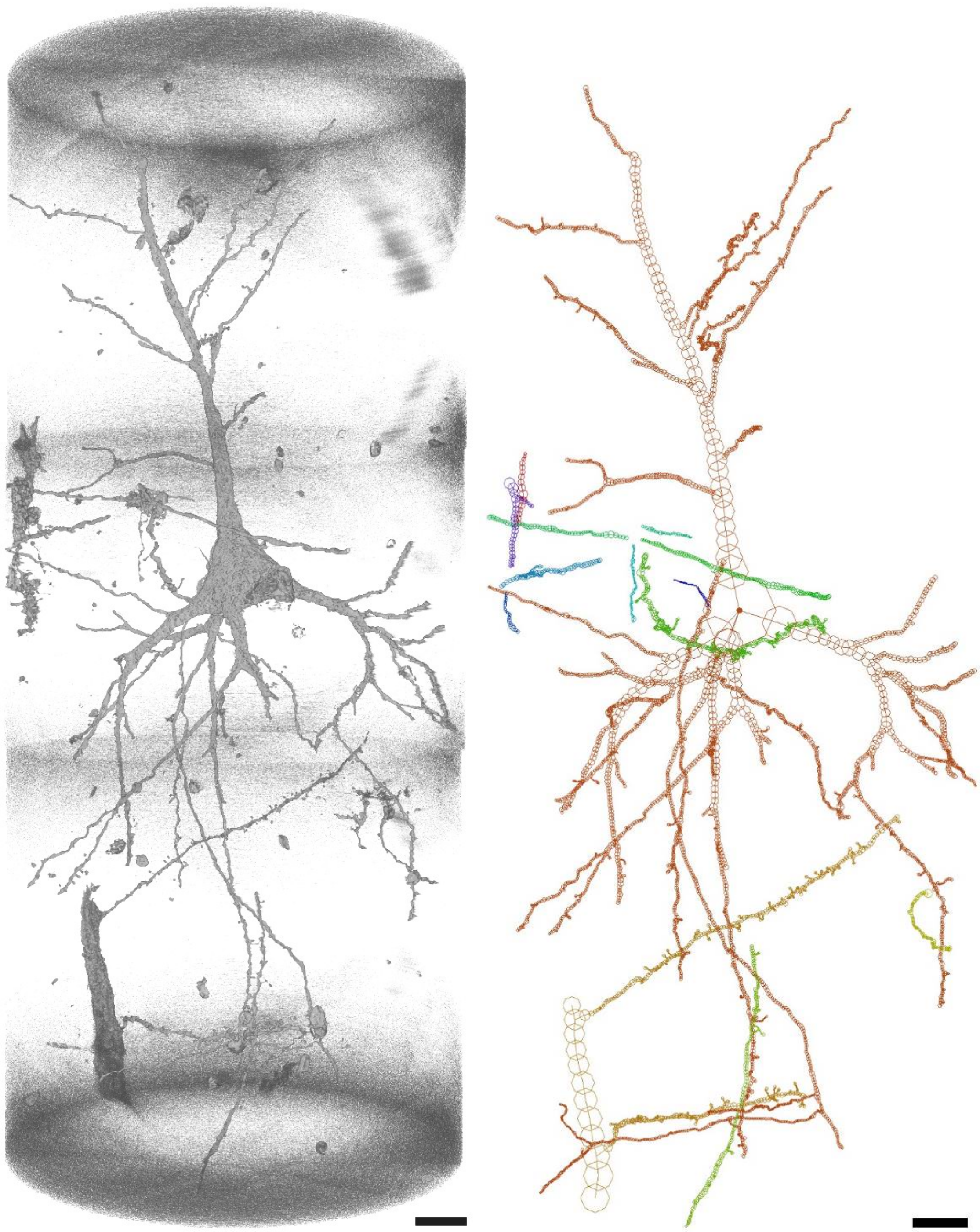

Supplementary Figure 3 (cont'd). Rendering of three-dimensional images of cerebral tissues and Cartesian coordinate models of the tissue structures. Scale bars: 10 μm. (**C**) Rendering of schizophrenia dataset S3A. (**D**) Model of S3A.

E **F**

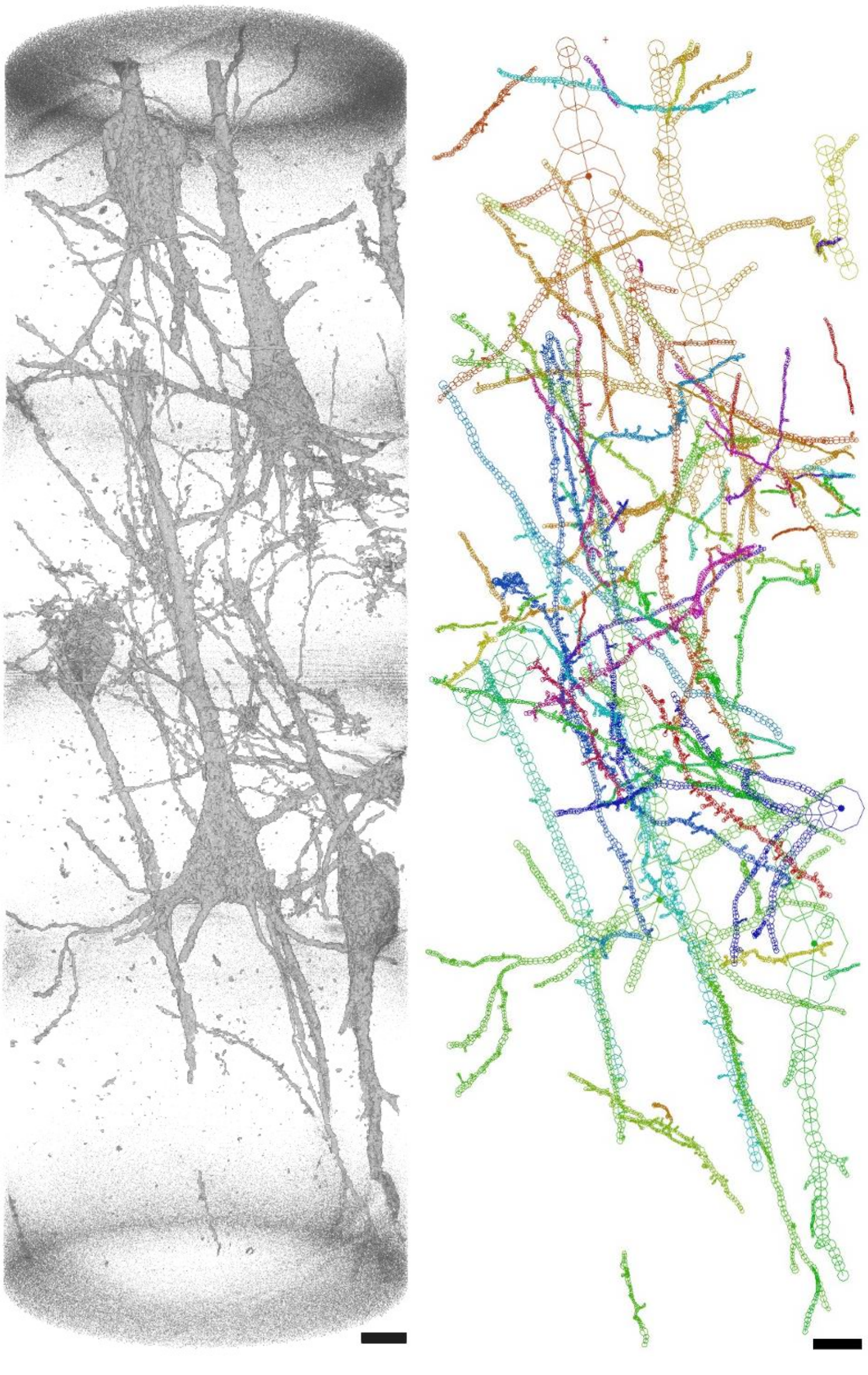

Supplementary Figure 3 (cont'd). Rendering of three-dimensional images of cerebral tissues and Cartesian coordinate models of the tissue structures. Scale bars: 10 µm. (**E**) Rendering of schizophrenia dataset S4D. (**F**) Model of S4D.

G **H**

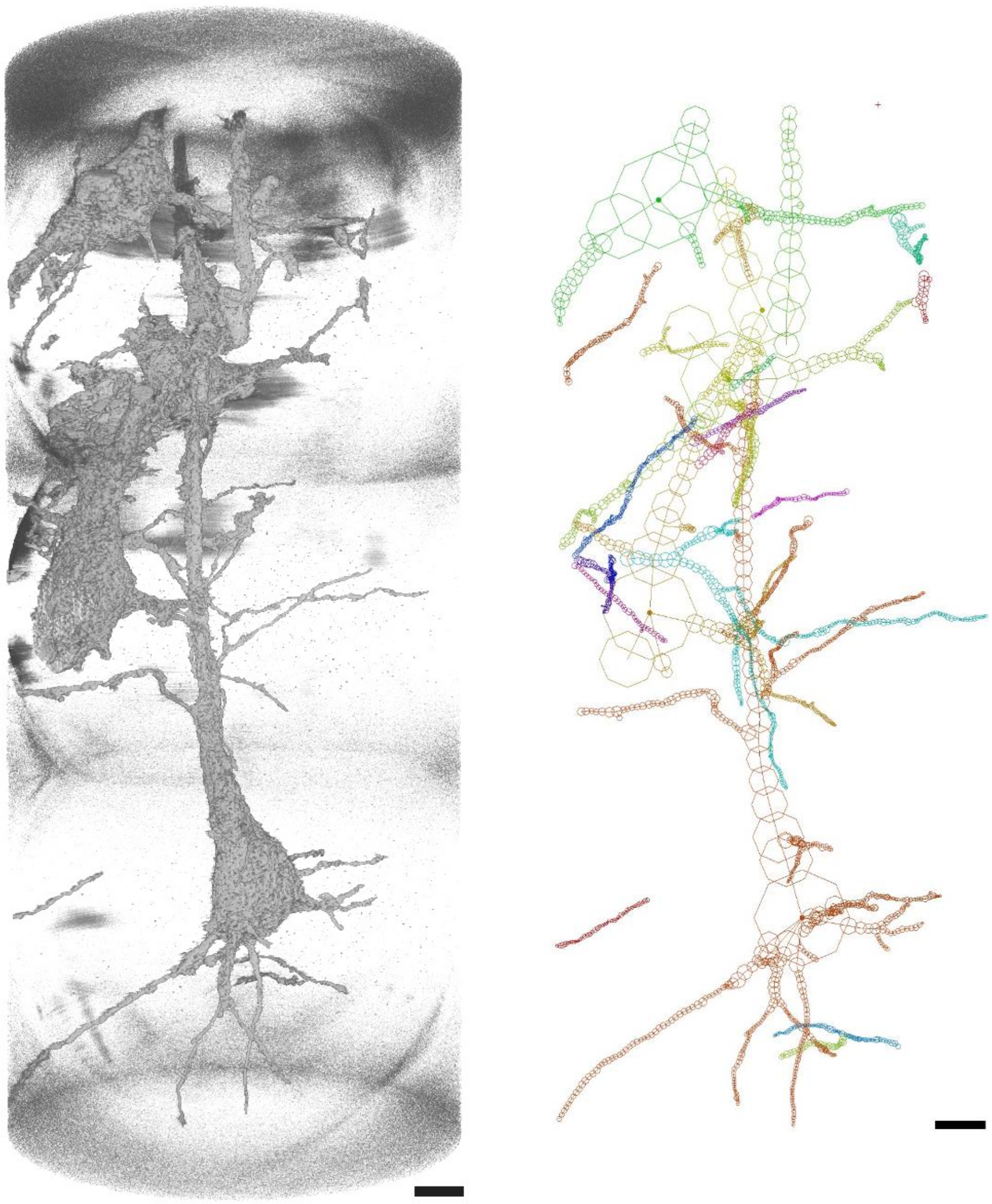

Supplementary Figure 3 (cont'd). Rendering of three-dimensional images of cerebral tissues and Cartesian coordinate models of the tissue structures. Scale bars: 10 µm. (G) Rendering of control dataset N1C. (**H**) Model of N1C.

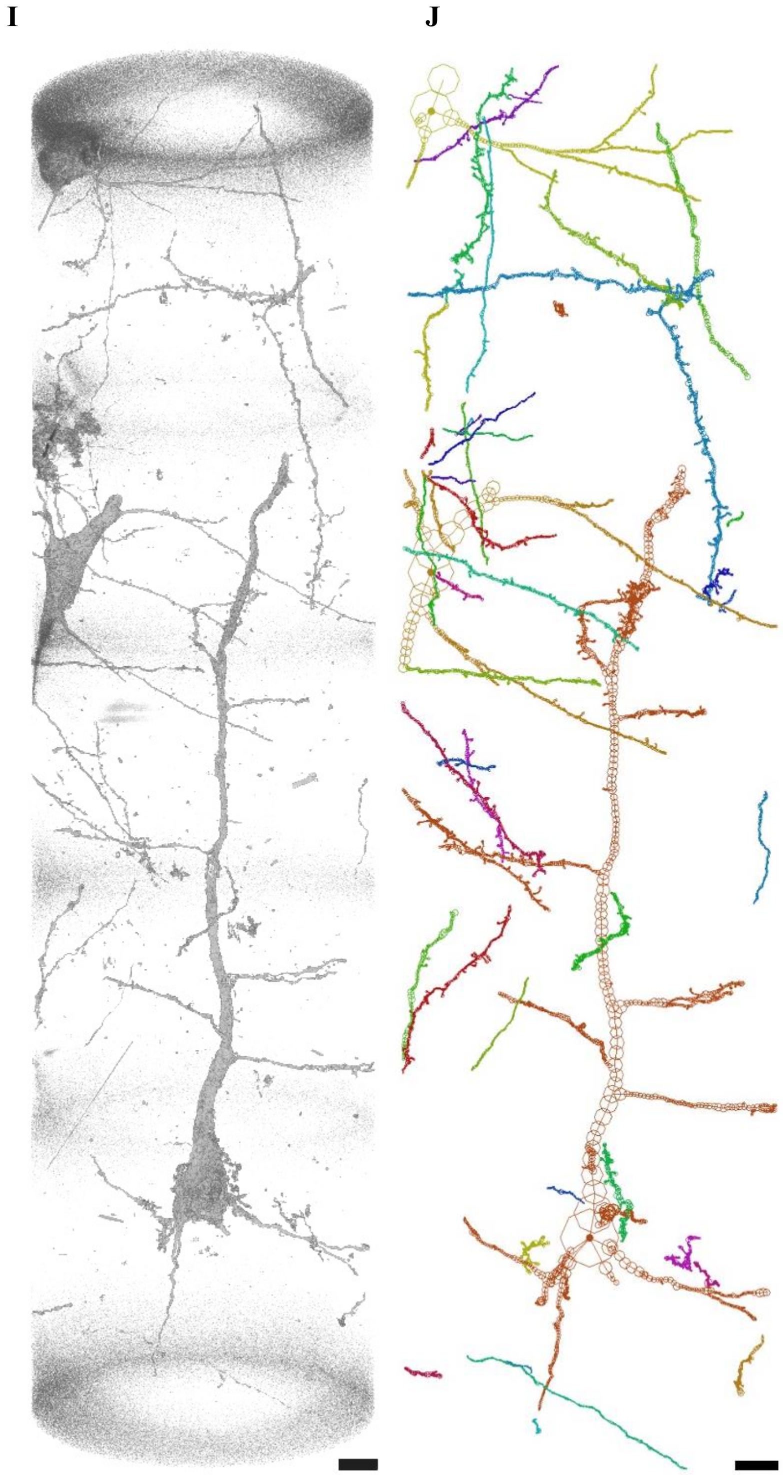

Supplementary Figure 3 (cont'd). Rendering of three-dimensional images of cerebral tissues and Cartesian coordinate models of the tissue structures. Scale bars: 10 µm. (**I**) Rendering of control dataset N2A. (**J**) Model of N2A.

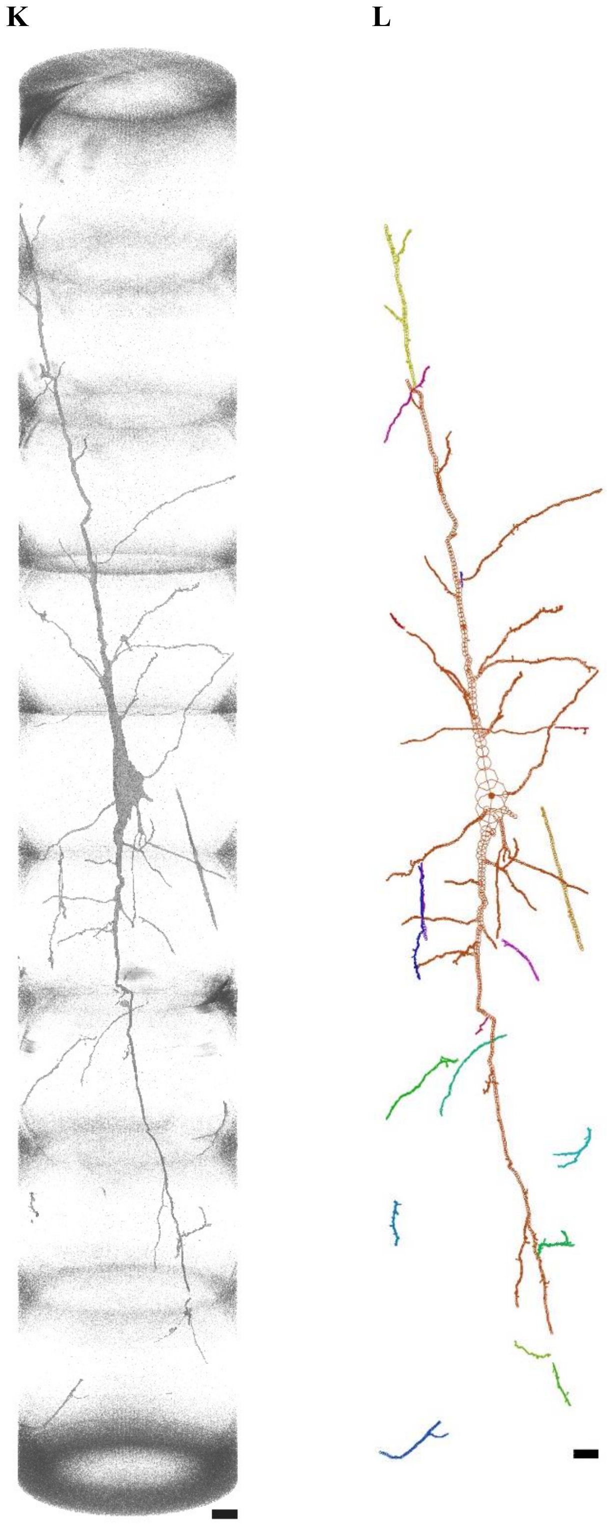

Supplementary Figure 3 (cont'd). Rendering of three-dimensional images of cerebral tissues and Cartesian coordinate models of the tissue structures. Scale bars: 10 µm. (**K**) Rendering of control dataset N3A. (**L**) Model of N3A.

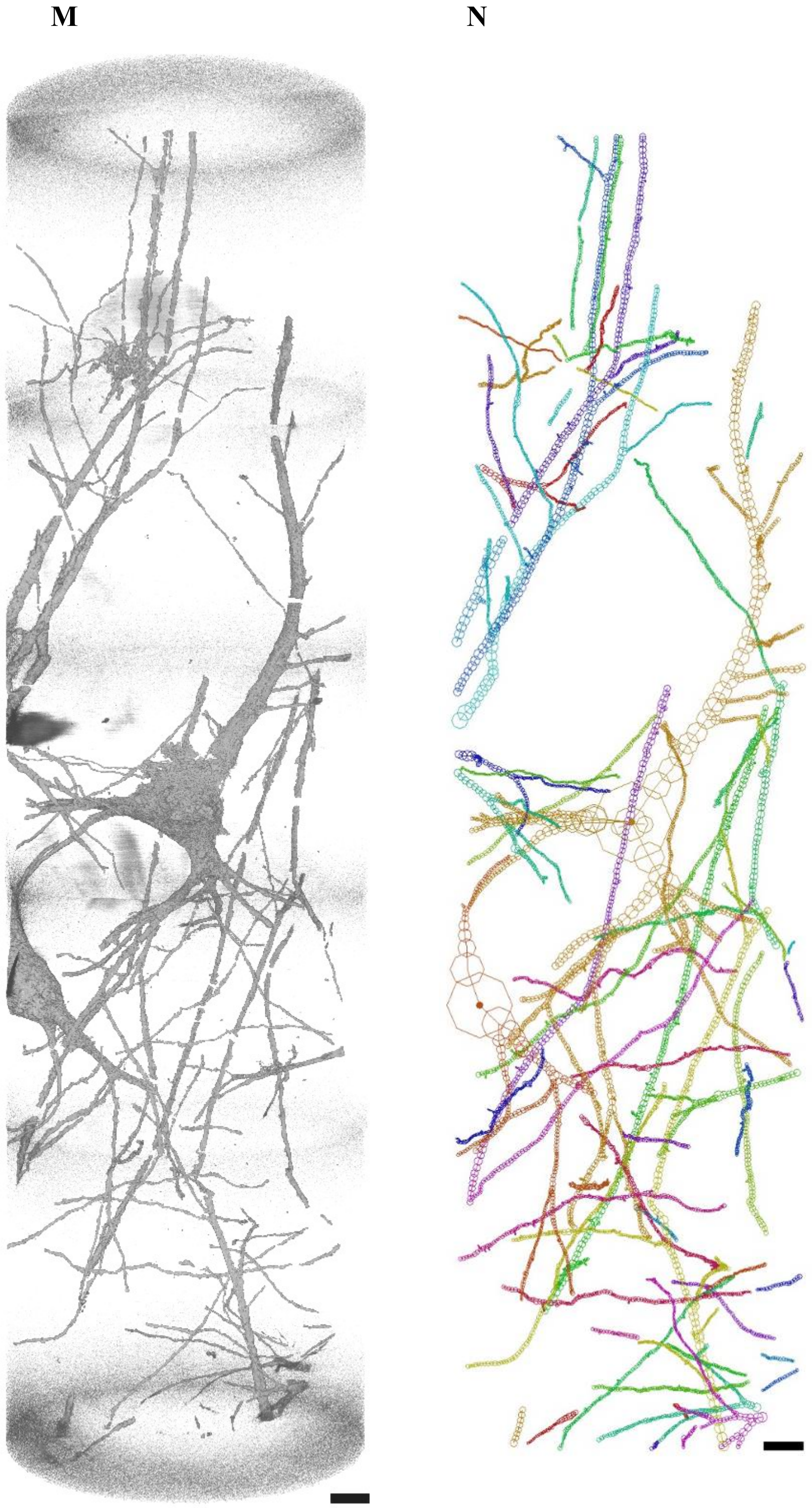

Supplementary Figure 3 (cont'd). Rendering of three-dimensional images of cerebral tissues and Cartesian coordinate models of the tissue structures. Scale bars: 10 µm. (**M**) Rendering of control dataset N4B. (**N**) Model of N4B.

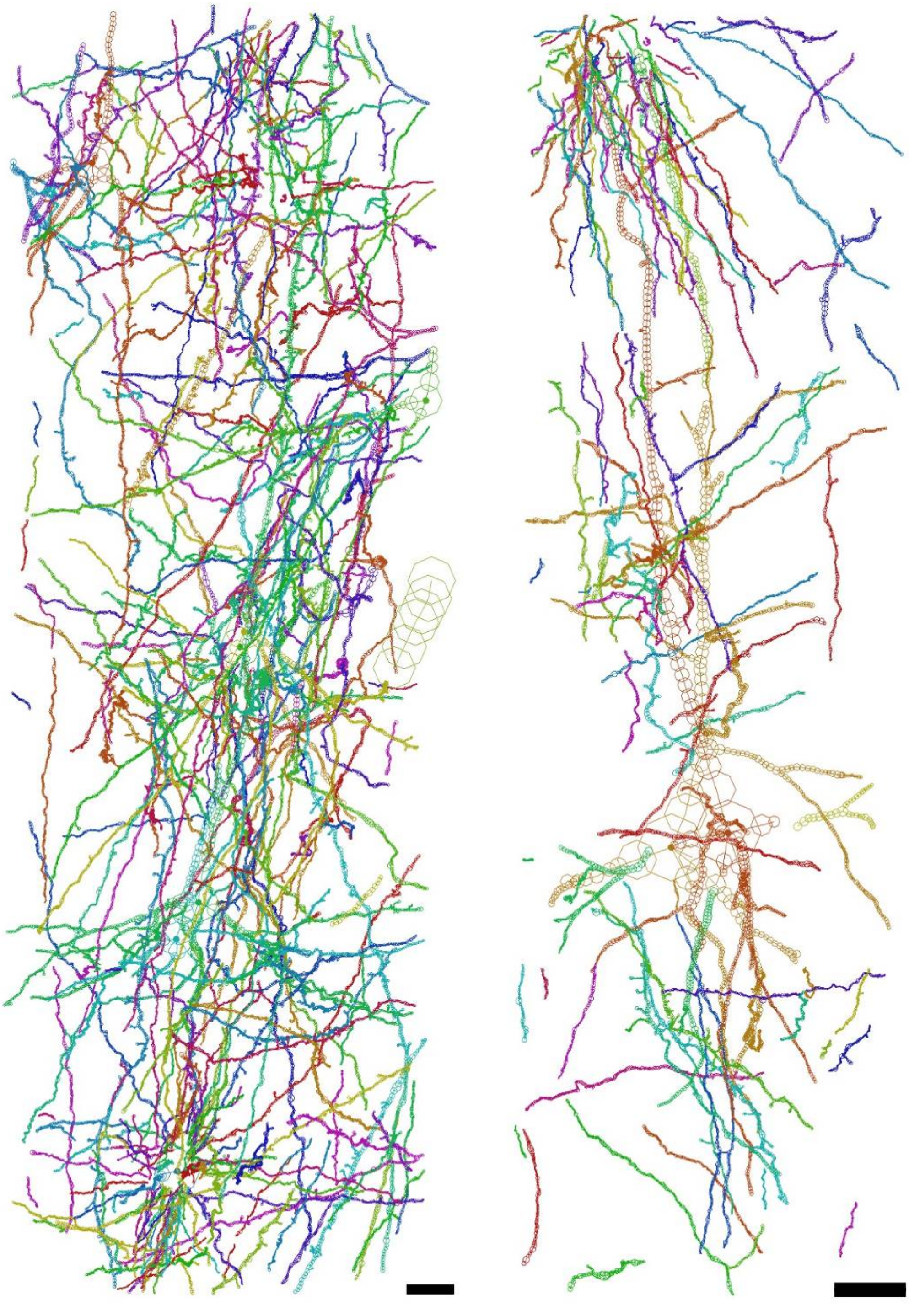

(**A**) S1B structure. (**B**) S1C structure.

Supplementary Figure 4. Cartesian coordinate models of schizophrenia case structures. The pial surface is toward the top. The models were drawn with the MCTrace software. Constituents of the models are color-coded. Nodes composing each constituent are indicated with octagons. Dots indicate somata nodes. Scale bars: 10 μm.

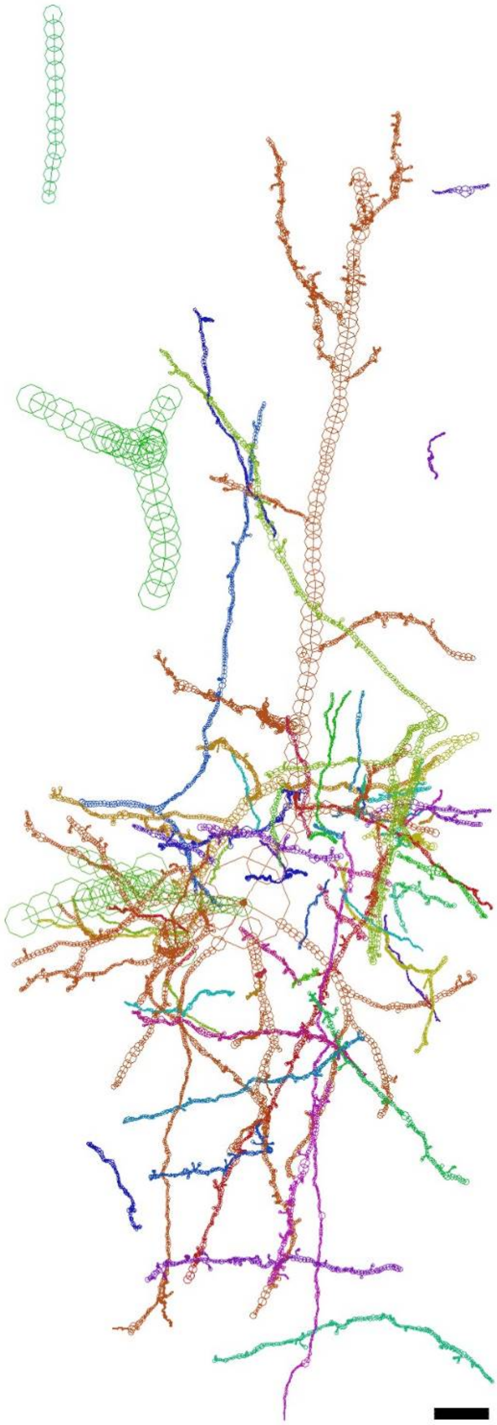

(**C**) S1D structure. (**D**) S2A structure.

Supplementary Figure 4 (cont'd). Cartesian coordinate models of schizophrenia case structures. The pial surface is toward the top. The models were drawn with the MCTrace software. Constituents of the models are color-coded. Nodes composing each constituent are indicated with octagons. Dots indicate somata nodes. Scale bars: 10 µm.

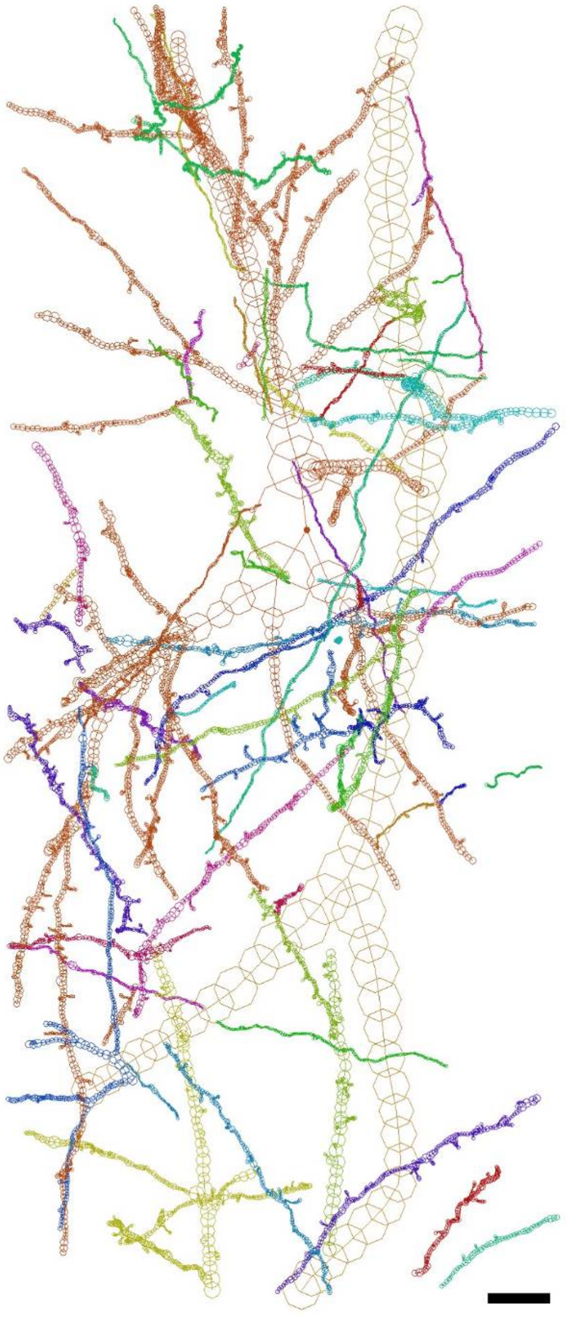

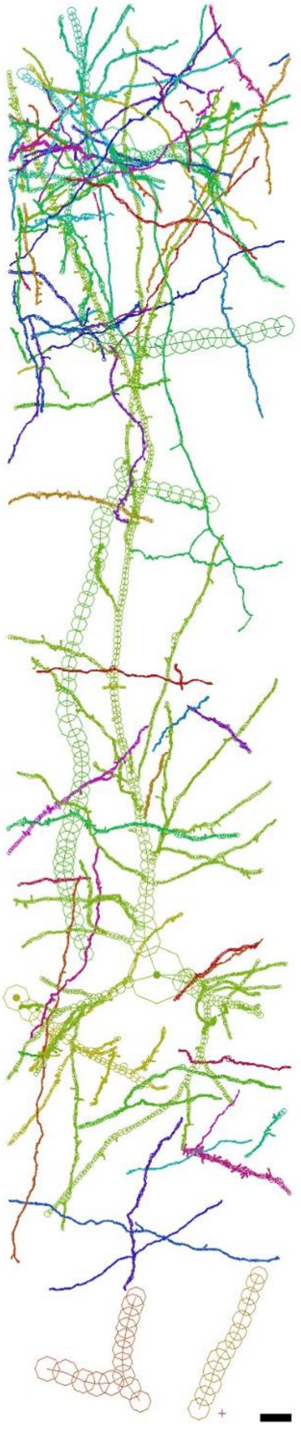

(**E**) S2C structure. (**F**) S2D structure.

Supplementary Figure 4 (cont'd). Cartesian coordinate models of schizophrenia case structures. The pial surface is toward the top. The models were drawn with the MCTrace software. Constituents of the models are color-coded. Nodes composing each constituent are indicated with octagons. Dots indicate somata nodes. Scale bars: 10 μm.

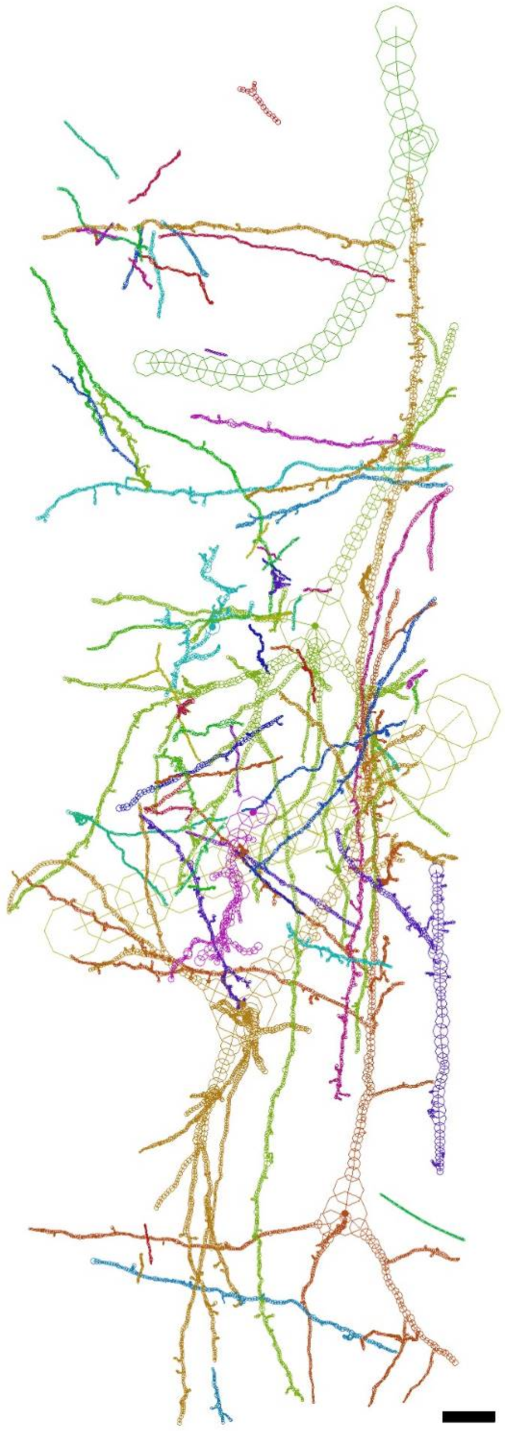

(**G**) S3B structure.

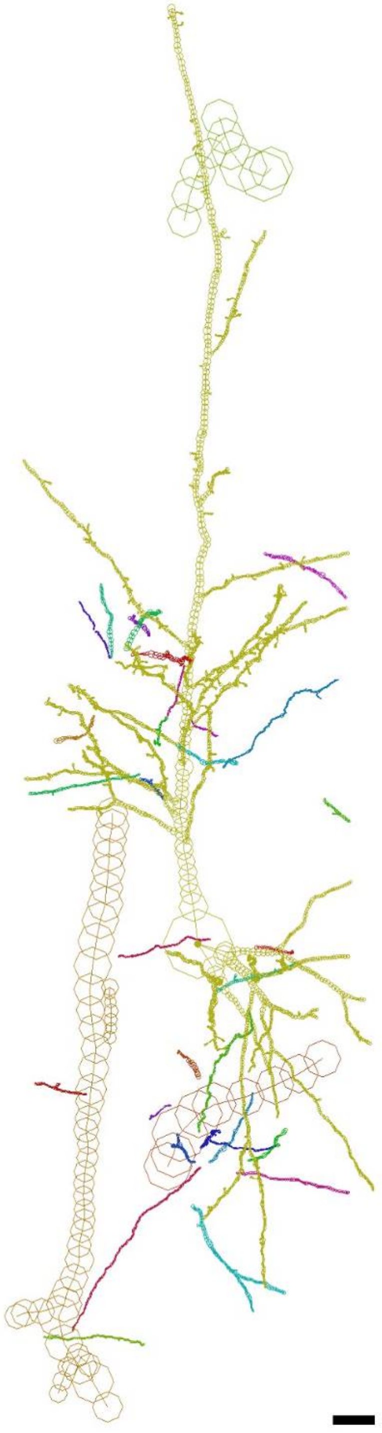

(**H**) S3C structure.

Supplementary Figure 4 (cont'd). Cartesian coordinate models of schizophrenia case structures. The pial surface is toward the top. The models were drawn with the MCTrace software. Constituents of the models are color-coded. Nodes composing each constituent are indicated with octagons. Dots indicate somata nodes. Scale bars: 10 μm.

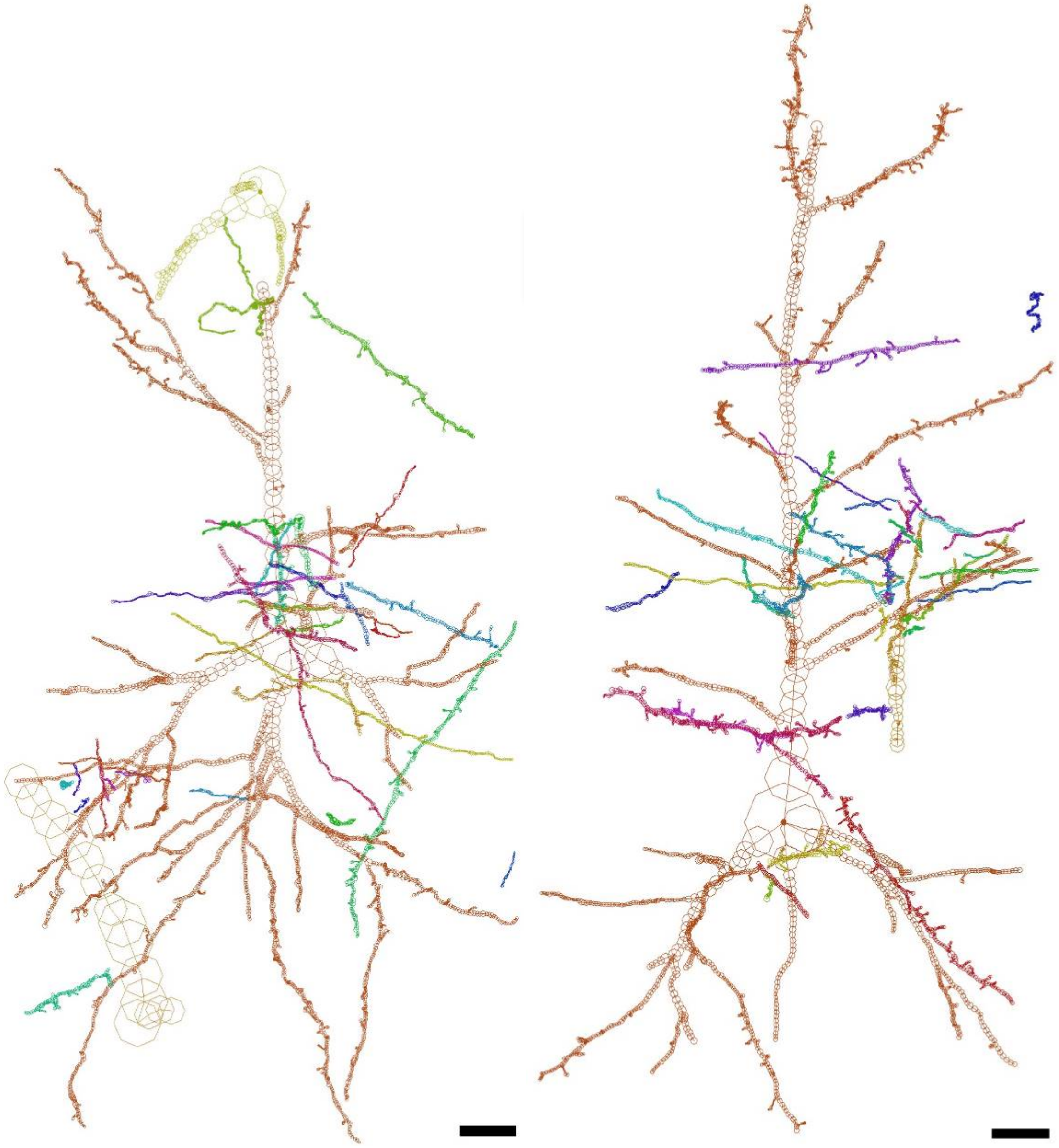

(**I**) S3D structure. (**J**) S3E structure.

Supplementary Figure 4 (cont'd). Cartesian coordinate models of schizophrenia case structures. The pial surface is toward the top. The models were drawn with the MCTrace software. Constituents of the models are color-coded. Nodes composing each constituent are indicated with octagons. Dots indicate somata nodes. Scale bars: 10 μm.

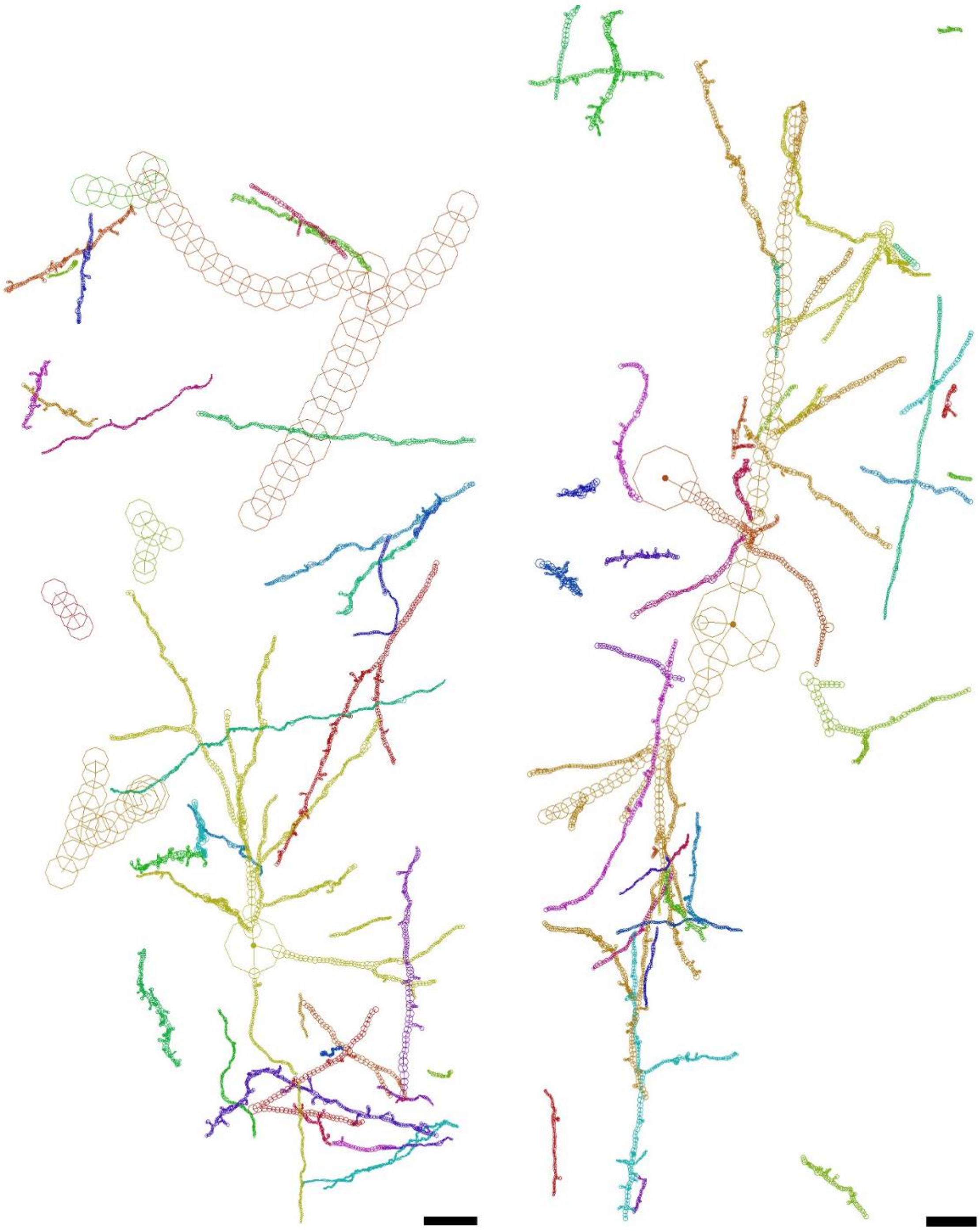

(**K**) S3F structure. (**L**) S4A structure.

Supplementary Figure 4 (cont'd). Cartesian coordinate models of schizophrenia case structures. The pial surface is toward the top. The models were drawn with the MCTrace software. Constituents of the models are color-coded. Nodes composing each constituent are indicated with octagons. Dots indicate somata nodes. Scale bars: 10 µm.

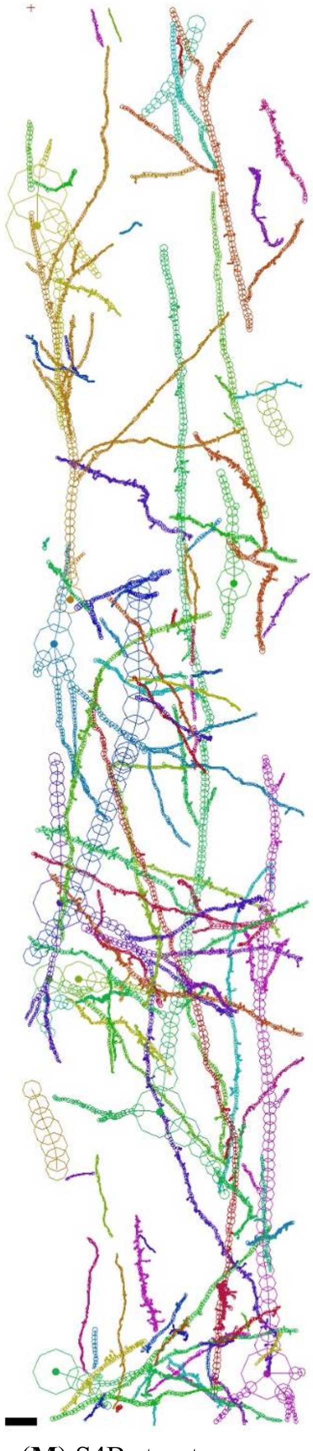

(**M**) S4B structure.

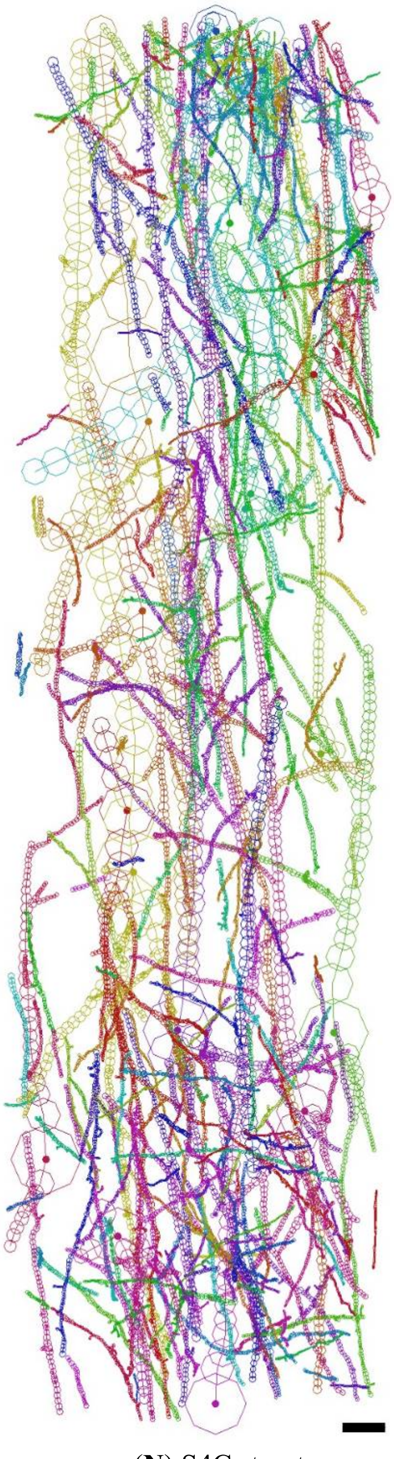

(**N**) S4C structure.

Supplementary Figure 4 (cont'd). Cartesian coordinate models of schizophrenia case structures. The pial surface is toward the top. The models were drawn with the MCTrace software. Constituents of the models are color-coded. Nodes composing each constituent are indicated with octagons. Dots indicate somata nodes. Scale bars: 10 µm.

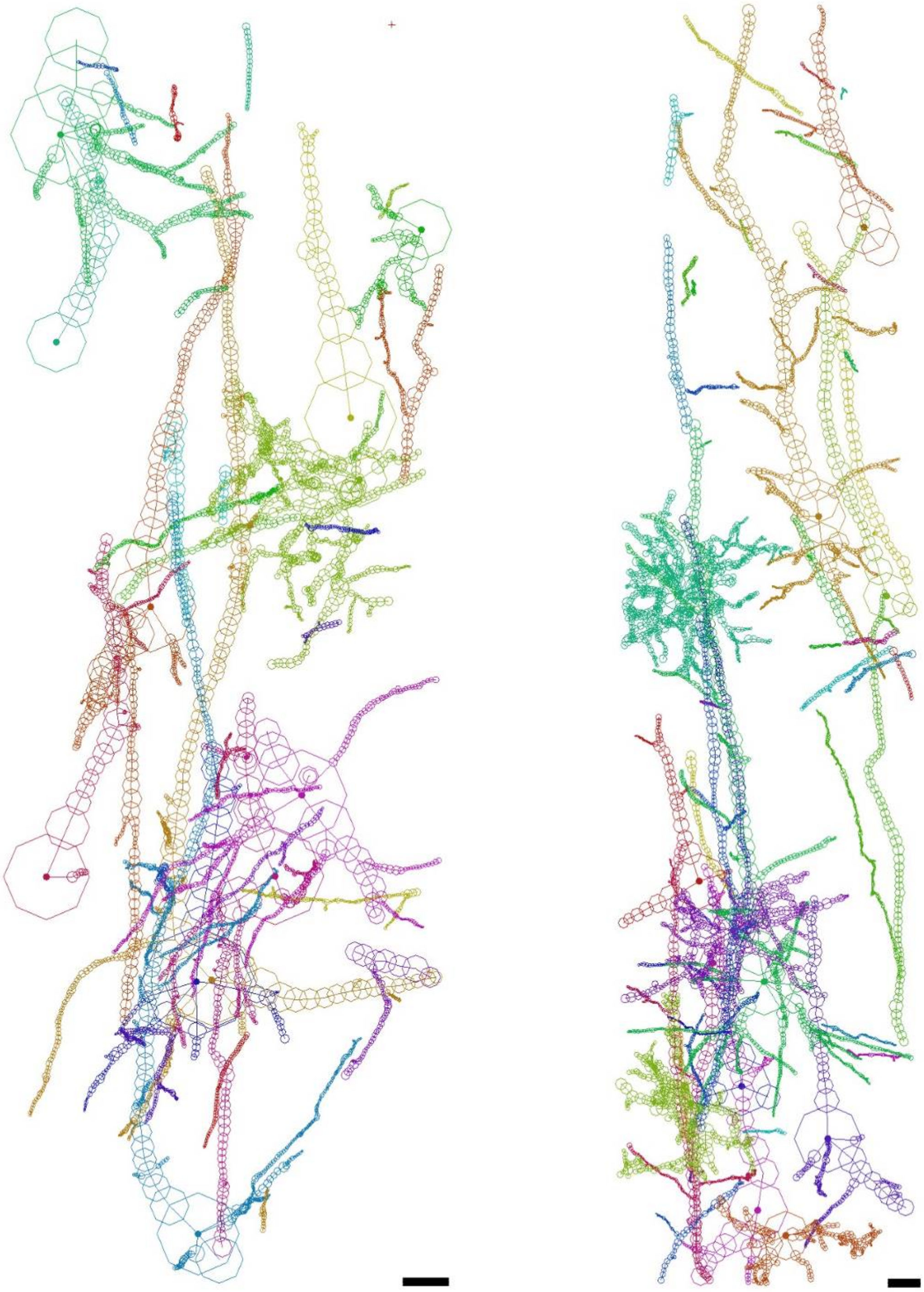

(**A**) N1A structure. (**B**) N1B structure.

Supplementary Figure 5. Cartesian coordinate models of control case structures. The pial surface is toward the top. The models were drawn with the MCTrace software. Constituents of the models are color-coded. Nodes composing each constituent are indicated with octagons. Dots indicate somata nodes. Scale bars: 10 μm.

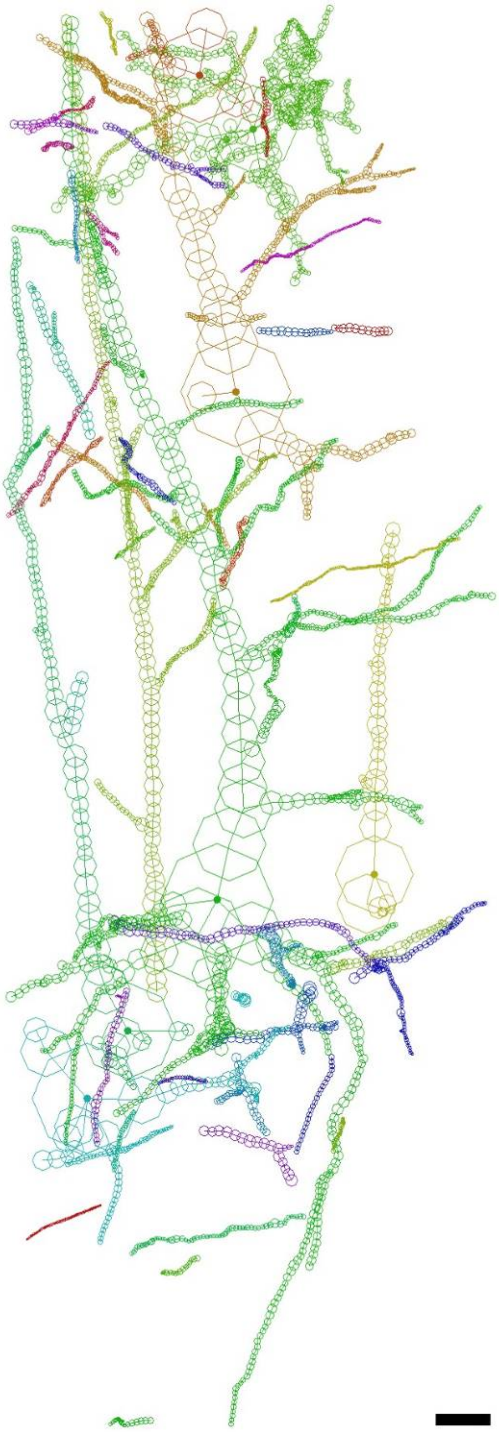

(**C**) N1D structure.

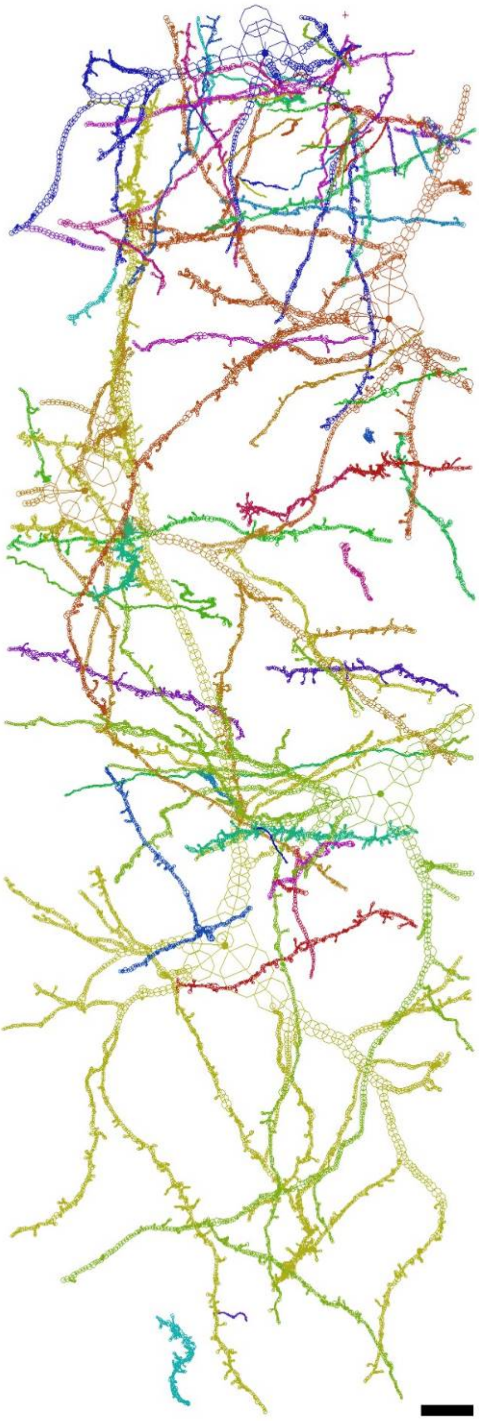

(**D**) N2B structure.

Supplementary Figure 5 (cont'd). Cartesian coordinate models of control case structures. The pial surface is toward the top. The models were drawn with the MCTrace software. Constituents of the models are color-coded. Nodes composing each constituent are indicated with octagons. Dots indicate somata nodes. Scale bars: 10 μm.

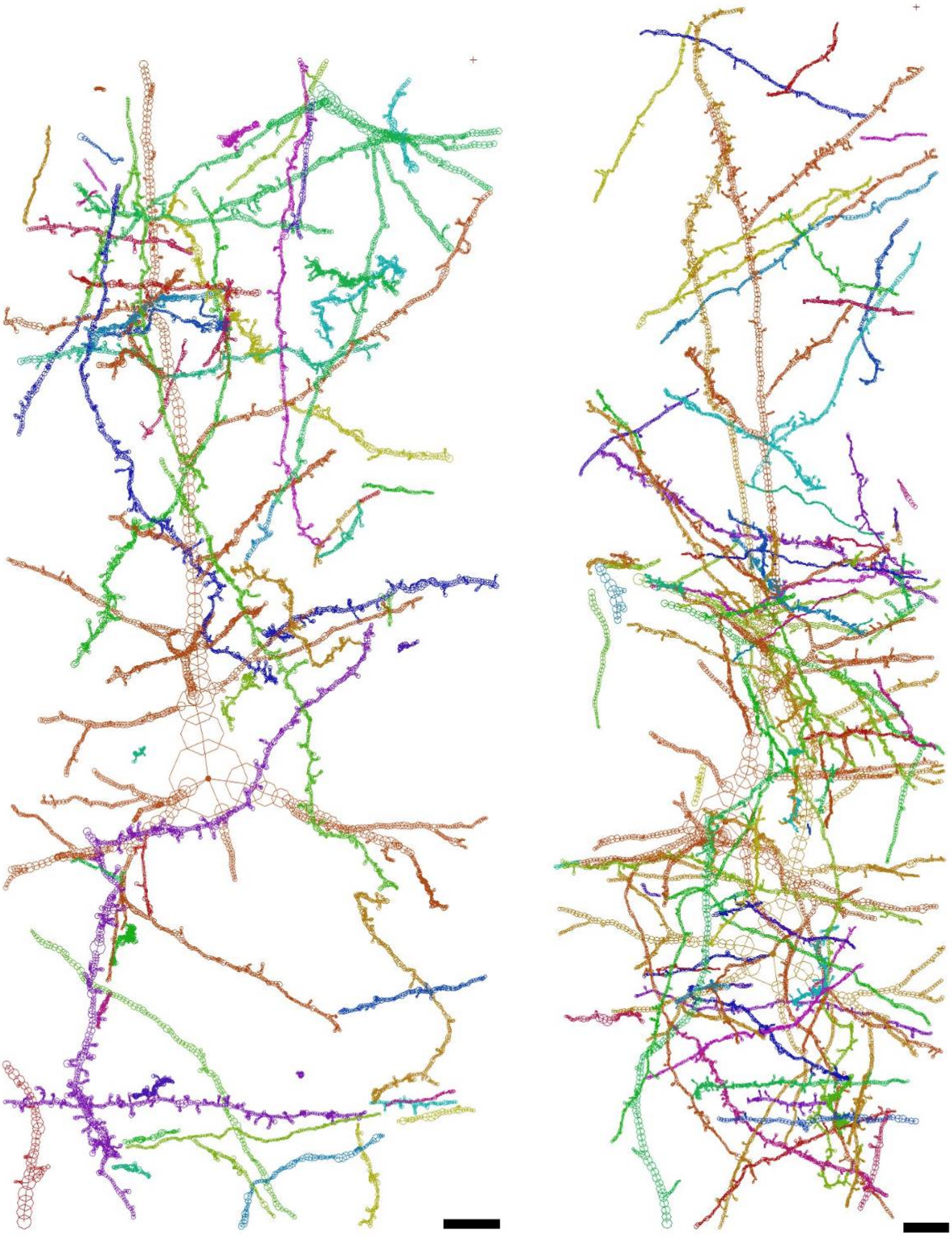

(**E**) N2C structure. (**F**) N2D structure.

Supplementary Figure 5 (cont'd). Cartesian coordinate models of control case structures. The pial surface is toward the top. The models were drawn with the MCTrace software. Constituents of the models are color-coded. Nodes composing each constituent are indicated with octagons. Dots indicate somata nodes. Scale bars: 10 μm.

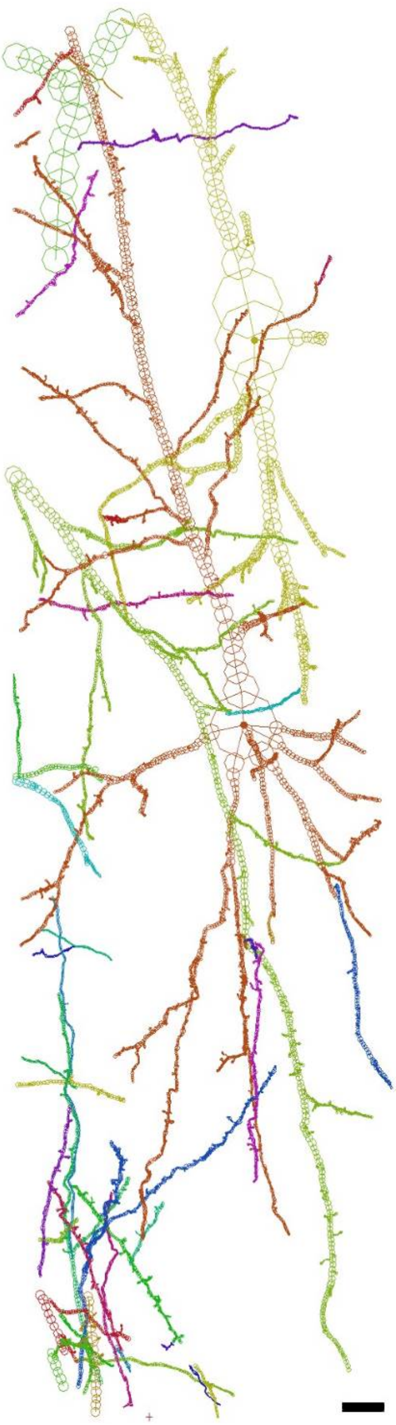

(**G**) N3B structure.

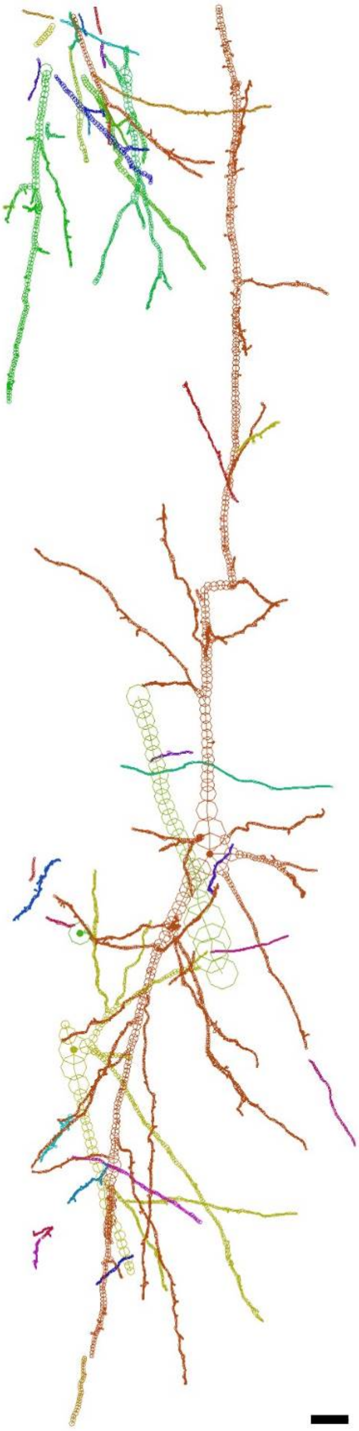

(**H**) N3C structure.

Supplementary Figure 5 (cont'd). Cartesian coordinate models of control case structures. The pial surface is toward the top. The models were drawn with the MCTrace software. Constituents of the models are color-coded. Nodes composing each constituent are indicated with octagons. Dots indicate somata nodes. Scale bars: 10 µm.

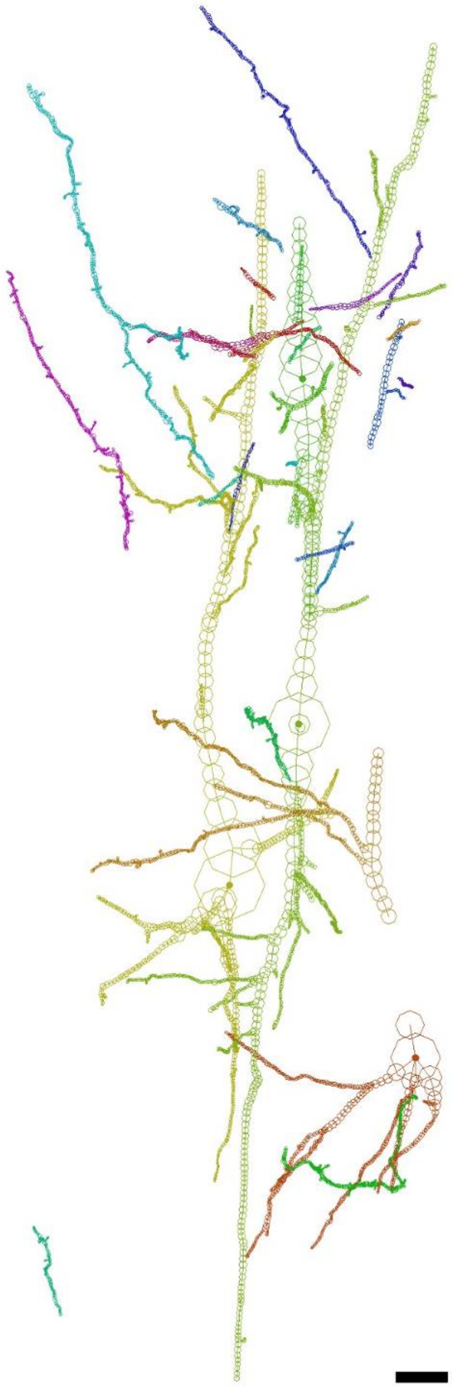

(**I**) N3D structure.

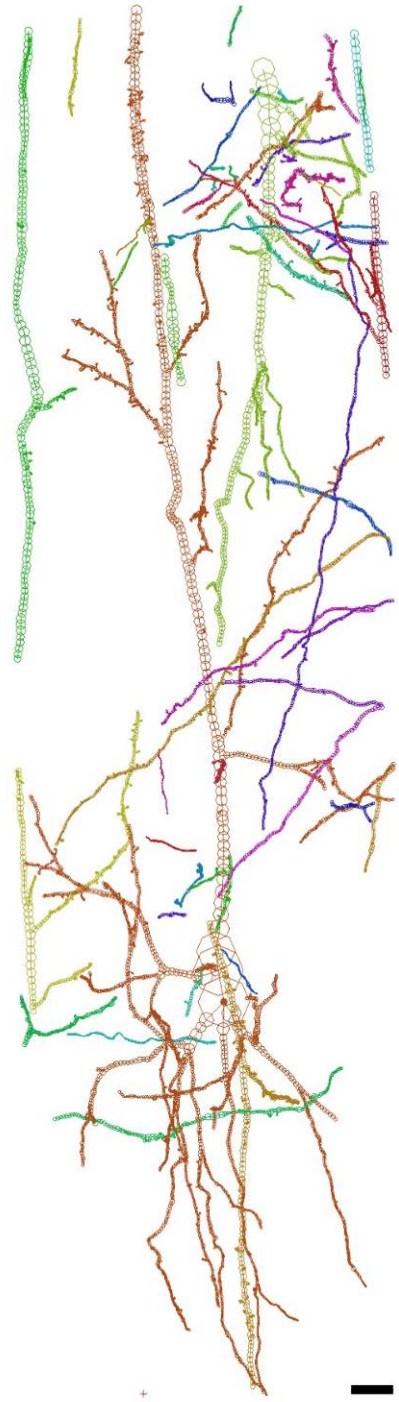

(**J**) N3E structure.

Supplementary Figure 5 (cont'd). Cartesian coordinate models of control case structures. The pial surface is toward the top. The models were drawn with the MCTrace software. Constituents of the models are color-coded. Nodes composing each constituent are indicated with octagons. Dots indicate somata nodes. Scale bars: 10 μm.

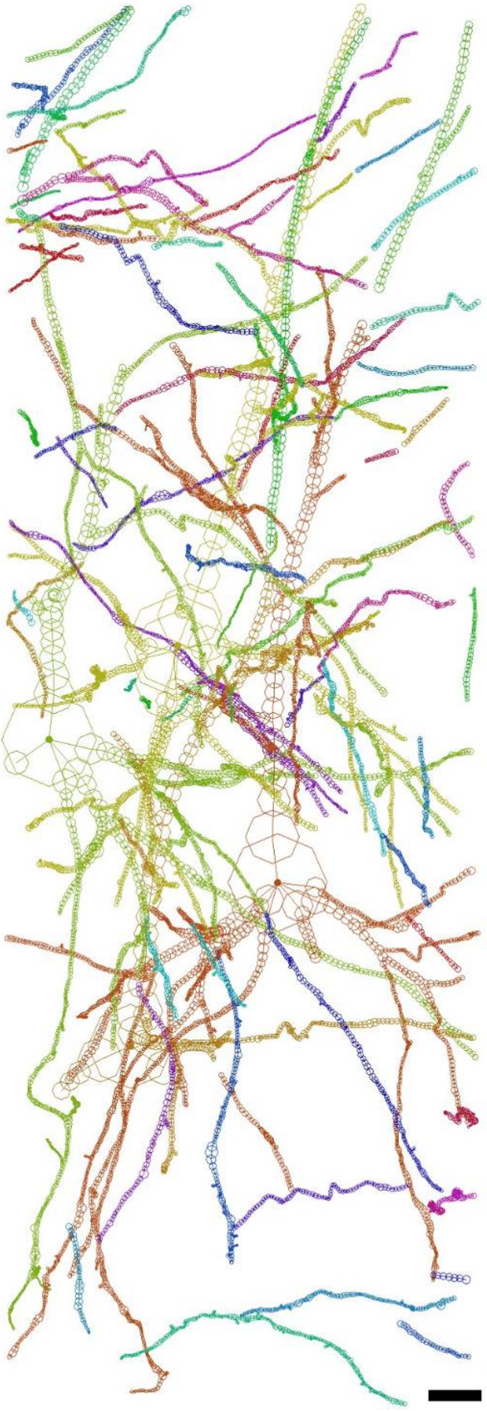

(**K**) N4A structure.

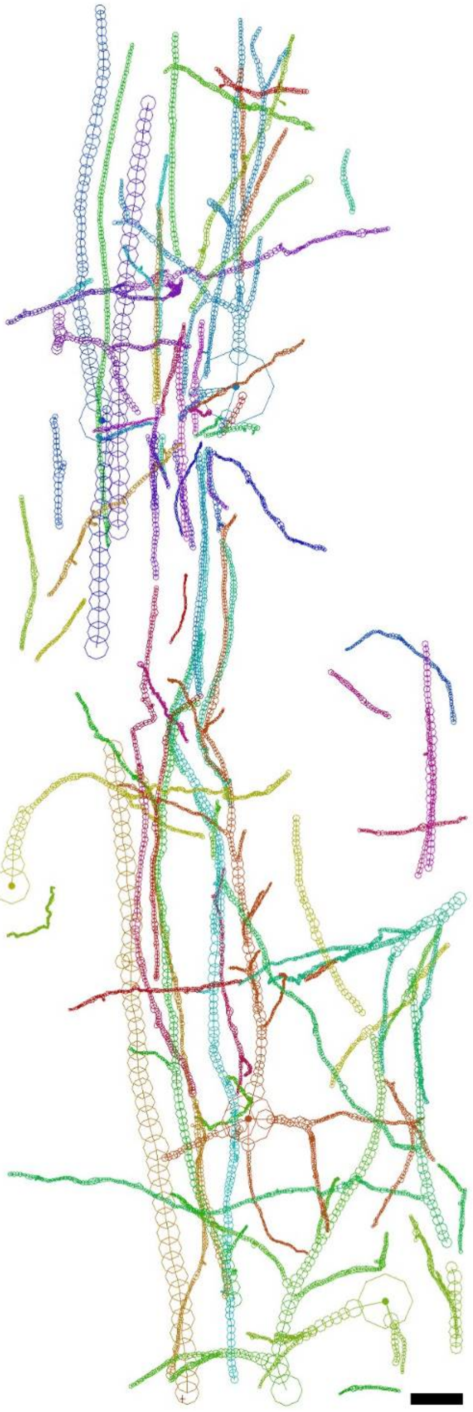

(**L**) N4C structure.

Supplementary Figure 5 (cont'd). Cartesian coordinate models of control case structures. The pial surface is toward the top. The models were drawn with the MCTrace software. Constituents of the models are color-coded. Nodes composing each constituent are indicated with octagons. Dots indicate somata nodes. Scale bars: 10 µm.

A

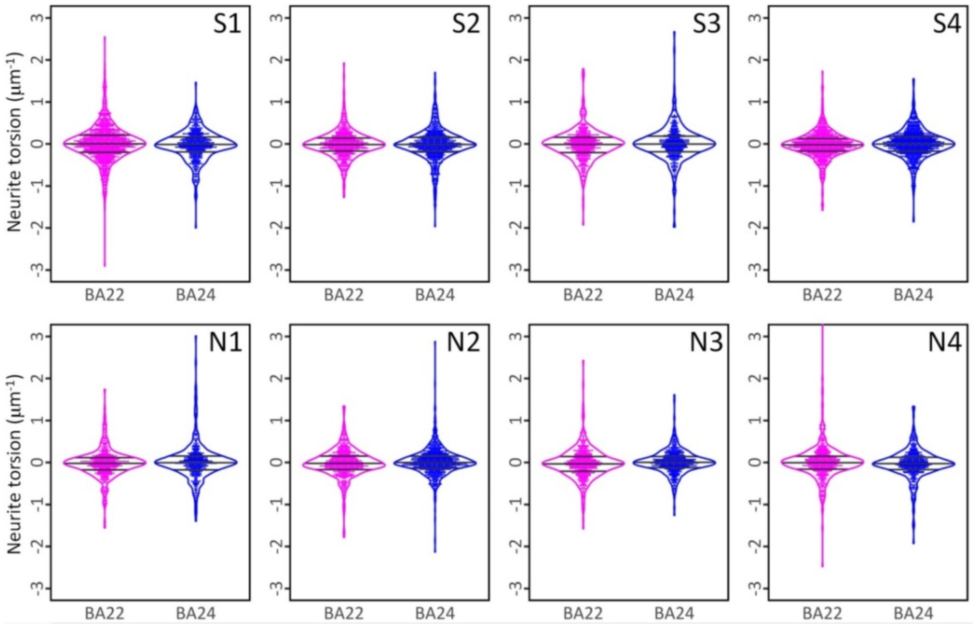

B

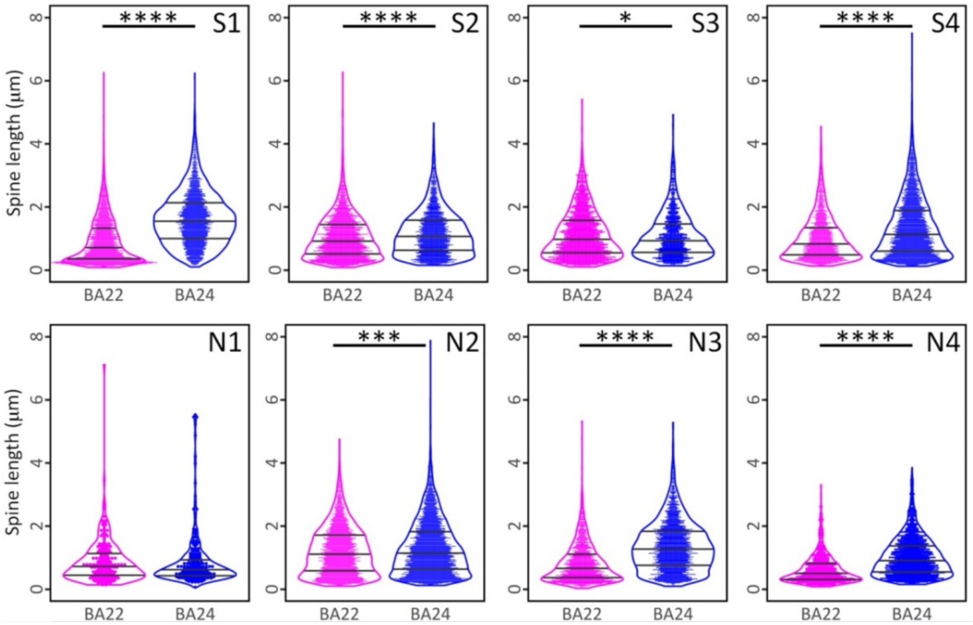

Supplementary Figure 6. Frequency distribution of neurite torsion (**A**) and spine length (**B**) of temporal (BA22) and prefrontal (BA24) cortexes of each case. Schizophrenia cases S1–S4 and controls N1–N4 are indicated with labels. A torsion outlier of BA22 of N4 (4.31 μm^{-1}) was omitted. The equality of distributions between BA22 and BA24 was examined using the Kolmogorov- Smirnov test, and their *p*-values were corrected with the Holm-Bonferroni method. ****$p < 10^{-8}$; ***$p < 10^{-4}$; *$p = 0.045$. Quartiles are indicated with bars. Dot size is adjusted for visibility.

Supplementary Table 4. Geometric parameters of neurites.

Case	Curvature (μm^{-1})		Torsion (μm^{-1})
	Total	Orphan neurite	
S1	1.08 (0.42) / 1073	1.19 (0.38) / 837	0.01 (0.42) / 1069
S2	0.56 (0.27) / 617	0.65 (0.25) / 399	-0.01 (0.31) / 615
S3	0.63 (0.30) / 505	0.78 (0.30) / 251	-0.02 (0.36) / 503
S4	0.36 (0.21) / 899	0.46 (0.21) / 497	-0.02 (0.31) / 878
N1	0.28 (0.16) / 477	0.35 (0.17) / 175	-0.02 (0.33) / 445
N2	0.58 (0.29) / 571	0.72 (0.30) / 306	-0.01 (0.33) / 564
N3	0.46 (0.22) / 506	0.52 (0.24) / 269	-0.02 (0.36) / 500
N4	0.35 (0.19) / 507	0.39 (0.19) / 308	0.01 (0.42) / 499

Values represent mean (sample standard deviation) / number of observations. S1–S4 are the schizophrenia cases, and N1–N4 are the control cases. The orphan neurite column represents statistics of neurites whose somata were out of the viewing field.

Supplementary Video caption

Supplementary Video 1. Schizophrenia S1A structure. The structure is zoomed in and out and rotated around the red neuron. Structural constituents are color-coded. Dots indicate soma positions. The pial surface is toward the top. Video frames were produced using the MCTrace software.
