# Supplementary material for: Structural diverseness of neurons between brain areas and between cases
Source: Transl Psychiatry. 2021 Jan 14;11:49. doi: 10.1038/s41398-020-01173-x (PMC7809156; doi:10.1038/s41398-020-01173-x)
Supplement: Supplementary file 3 — Supplementary Tables 1-3 [file 41398_2020_1173_MOESM3_ESM.pdf]

Supplementary Table 1. Statistics of structural analysis.

Case code	S1	S2	S3	S4	N1	N2	N3	N4
Gender	female	female	male	male	female	female	male	male
Age	56	70	64	69	58	72	62	65
Postmortem interval (hour)	2	7.5	21	7	85	3.5	8	17.5
Psychiatric record	schizophrenia	schizophrenia	schizophrenia	schizophrenia	no	no	no	no
BA22 structure								
Layer V depth (um)	1350-2000	1950-2750	1300-2050	1950-2850	1850-2900	1450-2150	2850-3900	1650-2200
Number of datasets	4	4	6	4	4	4	5	3
Number of model nodes	127928	58804	42114	47624	15703	67502	37420	25511
Number of constituents	777	327	246	459	162	270	200	228
Pyramidal neurons	13	6	7	30	28	10	10	8
Interneurons	0	0	1	2	0	0	1	1
Non-typed neurons	3	2	1	7	2	1	0	2
Orphan neurites	753	311	224	415	125	259	187	217
Gliaform cells	0	0	2	0	7	0	0	0
Blood capillaries	8	8	11	5	0	0	2	0
Total length (um) [1]	36929.1	24206.5	16345.4	28812.6	17304.5	21304.8	17173.0	16661.6
Pyramidal process (um)	9528.5	7735.5	8017.9	13181.0	8422.1	9000.0	8779.8	5673.3
Interneuron process (um)	0.0	0.0	467.9	285.4	0.0	0.0	7.1	225.1
Non-typed neuron process (um)	140.2	1254.3	89.0	715.8	225.4	470.1	0.0	210.6
Orphan neurite (um)	26895.2	14222.4	6651.9	14197.0	4748.2	11834.8	8213.3	10552.7
Gliaform cell process (um)	0.0	0.0	229.1	0.0	3908.8	0.0	0.0	0.0
Blood capillary (um)	365.2	994.2	889.5	433.3	0.0	0.0	172.8	0.0
Number of neurite segments	1075	636	511	920	517	581	518	523
Neurite curvature (um^{-1}) [2]	1.08 (0.42)	0.56 (0.27)	0.63 (0.30)	0.36 (0.21)	0.28 (0.16)	0.58 (0.29)	0.46 (0.22)	0.35 (0.19)
Neurite radius (um) [2]	0.24 (0.20)	0.44 (0.37)	0.38 (0.22)	0.71 (0.72)	1.16 (1.22)	0.45 (0.39)	0.48 (0.37)	0.61 (0.64)
Number of spines	4629	3746	2310	3532	283	5518	2423	877
Spine curvature (um^{-1}) [2]	1.75 (0.81)	1.31 (0.60)	1.42 (0.64)	1.12 (0.55)	0.94 (0.63)	1.43 (0.61)	1.36 (0.67)	1.13 (0.59)
Spine radius (um) [2]	0.18 (0.05)	0.22 (0.06)	0.20 (0.05)	0.24 (0.06)	0.30 (0.09)	0.21 (0.06)	0.21 (0.06)	0.24 (0.07)
Spine length (um) [2]	0.90 (0.70)	1.03 (0.67)	1.12 (0.74)	0.99 (0.67)	0.85 (0.66)	1.21 (0.76)	0.81 (0.59)	0.62 (0.44)
Spine density (um^{-1}) [3]	0.166	0.181	0.190	0.169	0.047	0.284	0.168	0.079
BA24 structure [4]								
Neurite curvature (um^{-1}) [2]	0.46 (0.28)	0.47 (0.32)	0.60 (0.34)	0.71 (0.36)	0.33 (0.22)	0.44 (0.21)	0.37 (0.21)	0.41 (0.23)
Neurite radius (um) [2]	0.66 (0.61)	0.63 (0.65)	0.58 (0.71)	0.39 (0.34)	1.06 (1.07)	0.53 (0.42)	0.71 (0.73)	0.59 (0.51)

Spine curvature (um^{-1}) [2]	1.13 (0.50)	1.15 (0.52)	1.19 (0.57)	1.16 (0.49)	0.80 (0.54)	1.18 (0.51)	1.16 (0.51)	1.12 (0.53)
Spine radius (um) [2]	0.23 (0.07)	0.21 (0.07)	0.22 (0.08)	0.21 (0.06)	0.29 (0.10)	0.22 (0.06)	0.20 (0.06)	0.22 (0.07)
Spine length (um) [2]	1.62 (0.82)	1.16 (0.70)	1.07 (0.69)	1.32 (0.92)	0.85 (0.80)	1.30 (0.84)	1.36 (0.77)	1.01 (0.60)
Spine density (um^{-1}) [3]	0.422	0.171	0.136	0.261	0.078	0.323	0.247	0.132

[1] Spine length is not included.

[2] Mean (standard deviation)

[3] Spine density = number of spines / total length of spiny dendrite

[4] Transl Psychiatry 9: 85 (2019)

Supplementary Table 2. Conditions of microtomography and nanotomography experiments.

Beamtime start date	2018.10.5	2018.11.16	2013.12.11 -2018.5.15
Facility	SPring-8	APS	SPring-8
Beamline	BL37XU	32-ID	BL20XU [1]
X-ray energy (keV)	8.0	8.0	12.0
Contrast	Absorption	Absorption	Absorption
Beam condenser	Sector zone plate	CRL[2] + capillary	-
Photon flux at the sample position (photons/mm^2/s) [3]	1.1×10^{14}	1.5×10^{14}	3.8×10^{11}
Outermost zone width (nm) / diameter (um) of Fresnel zone plate	100 / 310	50 / 180	-
Focal depth (um) [4]	258	65	-
Scintillator screen	P43 (Gd_2O_2S:Tb)	LuAG:Ce ($Lu_3Al_5O_{12}$:Ce)	LuAG:Ce or GAGG:Ce ($Gd_3Al_2Ga_3O_{12}$:Ce)
Pixel size (nm)	48.8	22.5	500
X-ray illumination (diameter in pixels)	1850	Full field	Full field
Viewing field (pixels) [5]	2048 x 2048	2448 x 2048	2048 x 2048
Maximum image width (um)	90	55	1024
Image dynamic range (bits) [6]	14	13	15-16
Number of sample frames per dataset	1800	1815	1800
Degrees per frame	0.100	0.099	0.100
Exposure time per frame (msec)	200	500	100-150
Data collection time (sec)	720	1200	400-550
Spatial resolution (nm)	200 [7] - 240 [8]	150 [7]- 200 [8]	1200 [7]

[1] Used for visualizing overall sample structures

[2] CRL: compound refractive lens

[3] Measured by using Al_2O_3:C dosimeters. The flux of BL20XU was measured on 2018.5.15 at the downstream hutch.

[4] Focal depth Δf was calculated with $\Delta f = \pm \lambda / (2\ NA^2)$ and $NA = \lambda / (2\Delta r_N)$,
where λ is the wavelength, NA is the numerical aperture, and Δr_N is the outermost zone width of Fresnel zone plate.

[5] Width x height

[6] Defined from the maximum intensity of flat field images

[7] Determined using three-dimensional square-wave test patterns. These estimates represent the resolution that the instruments can reach.

[8] Determined from the Fourier domain plot. These estimates represent the resolution of the sample image itself.

Supplementary Table 3. Statistics of datasets and Cartesian coordinate models. (**A**) Schizophrenia case S1.

Dataset name	S1A	S1B	S1C	S1D
Beamtime start date	2018.10.5	2018.10.5	2018.11.16	2018.11.16
Image size (pixel) [1]	1870 x 1860 x 5503	1860 x 1850 x 5506	1180 x 1200 x 3964	1190 x 1190 x 5906
Image size (um) [1]	91.3 x 90.8 x 268.5	90.8 x 90.3 x 268.7	53.1 x 54.0 x 178.4	53.6 x 53.6 x 265.8
Cortical depth of upper end (um)	1360	1390	1530	1560
Number of model nodes	43743	58031	16479	9675
Number of constituents	220	353	135	69
Pyramidal neurons	5	5	2	1
Interneurons	0	0	0	0
Non-typed neurons	1	2	0	0
Orphan neurites	210	345	133	65
Gliaform cells	0	0	0	0
Blood capillaries	4	1	0	3
Total length (um) [2]	12630.3	17343.9	4102.3	2852.6
Pyramidal process (um)	4656.5	2822.1	1003.9	1045.9
Interneuron process (um)	0.0	0.0	0.0	0.0
Non-typed neuron process (um)	109.1	31.1	0.0	0.0
Orphan neurite (um)	7617.8	14455.2	3098.3	1723.8
Gliaform cell process (um)	0.0	0.0	0.0	0.0
Blood capillary (um)	246.8	35.6	0.0	82.9
Number of neurite segments	315	459	189	112
Neurite curvature (um^{-1}) [3]	1.07 (0.37)	1.04 (0.36)	1.23 (0.56)	0.99 (0.40)
Neurite radius (um) [3]	0.23 (0.13)	0.24 (0.13)	0.26 (0.39)	0.24 (0.15)
Number of spines	2208	1862	396	163
Spine curvature (um^{-1}) [3]	1.79 (0.82)	1.60 (0.70)	2.15 (0.99)	2.07 (1.00)
Spine radius (um) [3]	0.18 (0.05)	0.19 (0.05)	0.14 (0.04)	0.15 (0.04)
Spine length (um) [3]	0.85 (0.70)	1.01 (0.70)	0.67 (0.57)	0.94 (0.75)
Spine density (um^{-1}) [4]	0.201	0.148	0.147	0.097

[1] Image width x height x number of slices. Image pixels of S1C and S1D datasets taken at the APS 32-ID beamline were averaged by 2 x 2 binning prior to the tomographic reconstruction.

[2] Spine length is not included.

[3] Mean (standard deviation)

[4] Spine density = number of spines / total length of spiny dendrite

Supplementary Table 3. Statistics of datasets and Cartesian coordinate models. (**B**) Schizophrenia case S2.

Dataset name	S2A	S2B	S2C	S2D
Beamtime start date	2018.10.5	2018.10.5	2018.10.5	2018.10.5
Image size (pixel) [1]	1870 x 1880 x 5505	1860 x 1880 x 4264	1870 x 1860 x 4263	1900 x 1880 x 9218
Image size (um) [1]	91.3 x 91.7 x 268.6	90.8 x 91.7 x 208.1	91.3 x 90.8 x 208.0	92.7 x 91.7 x 449.8
Cortical depth of upper end (um)	2580	2540	2440	2100
Number of model nodes	10723	18381	10996	18704
Number of constituents	69	90	64	104
Pyramidal neurons	1	2	1	2
Interneurons	0	0	0	0
Non-typed neurons	0	0	0	2
Orphan neurites	66	87	62	96
Gliaform cells	0	0	0	0
Blood capillaries	2	1	1	4
Total length (um) [2]	4058.6	7279.5	4510.5	8357.9
Pyramidal process (um)	1240.7	2708.8	1458.4	2327.6
Interneuron process (um)	0.0	0.0	0.0	0.0
Non-typed neuron process (um)	0.0	0.0	0.0	1254.3
Orphan neurite (um)	2557.7	4527.3	2772.3	4365.2
Gliaform cell process (um)	0.0	0.0	0.0	0.0
Blood capillary (um)	260.1	43.4	279.9	410.8
Number of neurite segments	119	190	127	200
Neurite curvature (um^{-1}) [3]	0.61 (0.28)	0.51 (0.22)	0.55 (0.27)	0.58 (0.30)
Neurite radius (um) [3]	0.40 (0.25)	0.48 (0.39)	0.45 (0.29)	0.43 (0.45)
Number of spines	722	1488	723	813
Spine curvature (um^{-1}) [3]	1.30 (0.59)	1.33 (0.60)	1.32 (0.61)	1.27 (0.58)
Spine radius (um) [3]	0.21 (0.06)	0.23 (0.06)	0.23 (0.07)	0.23 (0.07)
Spine length (um) [3]	1.07 (0.66)	0.98 (0.64)	1.12 (0.71)	1.02 (0.67)
Spine density (um^{-1}) [4]	0.204	0.222	0.196	0.119

[1] Image width x height x number of slices

[2] Spine length is not included.

[3] Mean (standard deviation)

[4] Spine density = number of spines / total length of spiny dendrite

Supplementary Table 3. Statistics of datasets and Cartesian coordinate models. (**C**) Schizophrenia case S3.

Dataset name	S3A	S3B	S3C	S3D	S3E	S3F
Beamtime start date	2018.10.5	2018.10.5	2018.10.5	2018.10.5	2018.10.5	2018.10.5
Image size (pixel) [1]	1860 x 1860 x 4266	1860 x 1880 x 5494	1870 x 1880 x 7981	1860 x 1850 x 4267	1860 x 1860 x 5497	1850 x 1850 x 4260
Image size (um) [1]	90.8 x 90.8 x 208.2	90.8 x 91.7 x 268.1	91.3 x 91.7 x 389.5	90.8 x 90.3 x 208.2	90.8 x 90.8 x 268.3	90.3 x 90.3 x 207.9
Cortical depth of upper end (um)	1490	1540	1520	1750	1740	1840
Number of model nodes	5141	12643	5591	7363	6257	5119
Number of constituents	14	75	37	39	40	41
Pyramidal neurons	1	3	1	1	1	0
Interneurons	0	0	0	0	0	1
Non-typed neurons	0	0	0	1	0	0
Orphan neurites	13	68	33	36	39	35
Gliaform cells	0	2	0	0	0	0
Blood capillaries	0	2	3	1	0	5
Total length (um) [2]	2153.5	4979.9	2539.8	2649.3	2036.3	1986.6
Pyramidal process (um)	1602.4	2478.7	1501.8	1415.9	1019.1	0.0
Interneuron process (um)	0.0	0.0	0.0	0.0	0.0	467.9
Non-typed neuron process (um)	0.0	0.0	0.0	89.0	0.0	0.0
Orphan neurite (um)	551.1	2024.8	711.3	1079.8	1017.2	1267.7
Gliaform cell process (um)	0.0	229.1	0.0	0.0	0.0	0.0
Blood capillary (um)	0.0	247.2	326.7	64.7	0.0	250.9
Number of neurite segments	61	162	74	86	73	55
Neurite curvature (um^{-1}) [3]	0.54 (0.25)	0.66 (0.29)	0.65 (0.27)	0.65 (0.35)	0.60 (0.30)	0.63 (0.27)
Neurite radius (um) [3]	0.45 (0.30)	0.37 (0.21)	0.35 (0.15)	0.38 (0.25)	0.38 (0.24)	0.33 (0.11)
Number of spines	299	620	274	370	485	262
Spine curvature (um^{-1}) [3]	1.40 (0.68)	1.48 (0.69)	1.36 (0.57)	1.44 (0.63)	1.41 (0.59)	1.35 (0.62)
Spine radius (um) [3]	0.19 (0.05)	0.20 (0.05)	0.20 (0.06)	0.20 (0.05)	0.20 (0.05)	0.22 (0.06)
Spine length (um) [3]	1.03 (0.70)	1.06 (0.73)	1.25 (0.71)	1.12 (0.76)	1.20 (0.77)	1.11 (0.70)
Spine density (um^{-1}) [4]	0.171	0.175	0.154	0.187	0.285	0.190

[1] Image width x height x number of slices

[2] Spine length is not included.

[3] Mean (standard deviation)

[4] Spine density = number of spines / total length of spiny dendrite

Supplementary Table 3. Statistics of datasets and Cartesian coordinate models. (**D**) Schizophrenia case S4.

Dataset name	S4A	S4B	S4C	S4D
Beamtime start date	2018.10.5	2018.10.5	2018.10.5	2018.10.5
Image size (pixel) [1]	1850 x 1850 x 5493	1860 x 1860 x 9193	1860 x 1860 x 6726	1850 x 1850 x 5503
Image size (um) [1]	90.3 x 90.3 x 268.1	90.8 x 90.8 x 448.6	90.8 x 90.8 x 328.2	90.3 x 90.3 x 268.5
Cortical depth of upper end (um)	2210	2180	1990	2530
Number of model nodes	3674	12459	21816	9675
Number of constituents	42	102	252	63
Pyramidal neurons	1	7	18	4
Interneurons	0	1	1	0
Non-typed neurons	1	3	2	1
Orphan neurites	40	88	230	57
Gliaform cells	0	0	0	0
Blood capillaries	0	3	1	1
Total length (um) [2]	1857.4	7050.0	14484.8	5420.4
Pyramidal process (um)	551.4	2648.6	7368.7	2612.3
Interneuron process (um)	0.0	136.6	148.8	0.0
Non-typed neuron process (um)	87.0	165.6	197.8	265.4
Orphan neurite (um)	1219.0	3905.0	6568.3	2504.8
Gliaform cell process (um)	0.0	0.0	0.0	0.0
Blood capillary (um)	0.0	194.2	201.3	37.8
Number of neurite segments	73	184	499	164
Neurite curvature (um^{-1}) [3]	0.39 (0.17)	0.35 (0.18)	0.37 (0.23)	0.33 (0.18)
Neurite radius (um) [3]	0.65 (0.81)	0.73 (0.65)	0.71 (0.79)	0.69 (0.52)
Number of spines	256	1231	1149	896
Spine curvature (um^{-1}) [3]	1.15 (0.51)	1.15 (0.56)	1.14 (0.58)	1.07 (0.52)
Spine radius (um) [3]	0.24 (0.06)	0.24 (0.06)	0.24 (0.06)	0.25 (0.06)
Spine length (um) [3]	1.05 (0.70)	1.04 (0.68)	0.79 (0.52)	1.16 (0.75)
Spine density (um^{-1}) [4]	0.194	0.207	0.119	0.226

[1] Image width x height x number of slices

[2] Spine length is not included.

[3] Mean (standard deviation)

[4] Spine density = number of spines / total length of spiny dendrite

Supplementary Table 3. Statistics of datasets and Cartesian coordinate models. (**E**) Control case N1.

Dataset name	N1A	N1B	N1C	N1D
Beamtime start date	2018.10.5	2018.10.5	2018.10.5	2018.10.5
Image size (pixel) [1]	1870 x 1870 x 5494	1880 x 1890 x 7958	1890 x 1910 x 4266	1900 x 1900 x 5516
Image size (um) [1]	91.3 x 91.3 x 268.1	91.7 x 92.2 x 388.4	92.2 x 93.2 x 208.2	92.7 x 92.7 x 269.2
Cortical depth of upper end (um)	2240	2080	1990	1840
Number of model nodes	4090	5791	2104	3718
Number of constituents	38	50	23	51
Pyramidal neurons	10	9	4	5
Interneurons	0	0	0	0
Non-typed neurons	0	0	1	1
Orphan neurites	26	37	18	44
Gliaform cells	2	4	0	1
Blood capillaries	0	0	0	0
Total length (um) [2]	4336.6	6972.1	2081.5	3914.3
Pyramidal process (um)	2540.2	2772.9	1201.0	1908.0
Interneuron process (um)	0.0	0.0	0.0	0.0
Non-typed neuron process (um)	0.0	0.0	170.9	54.5
Orphan neurite (um)	963.7	1698.3	709.7	1376.5
Gliaform cell process (um)	832.6	2500.9	0.0	575.3
Blood capillary (um)	0.0	0.0	0.0	0.0
Number of neurite segments	140	150	88	139
Neurite curvature (um^{-1}) [3]	0.27 (0.16)	0.29 (0.18)	0.27 (0.15)	0.27 (0.15)
Neurite radius (um) [3]	1.24 (1.29)	1.09 (1.08)	1.23 (1.37)	1.12 (1.18)
Number of spines	112	72	37	62
Spine curvature (um^{-1}) [3]	1.03 (0.65)	0.91 (0.58)	0.78 (0.51)	0.95 (0.73)
Spine radius (um) [3]	0.30 (0.09)	0.30 (0.09)	0.29 (0.09)	0.31 (0.10)
Spine length (um) [3]	0.84 (0.55)	1.05 (0.94)	0.72 (0.32)	0.74 (0.55)
Spine density (um^{-1}) [4]	0.066	0.038	0.042	0.039

[1] Image width x height x number of slices

[2] Spine length is not included.

[3] Mean (standard deviation)

[4] Spine density = number of spines / total length of spiny dendrite

Supplementary Table 3. Statistics of datasets and Cartesian coordinate models. (**F**) Control case N2.

Dataset name	N2A	N2B	N2C	N2D
Beamtime start date	2018.10.5	2018.10.5	2018.10.5	2018.10.5
Image size (pixel) [1]	1820 x 1820 x 6730	1870 x 1860 x 5494	1850 x 1860 x 4258	1850 x 1850 x 5486
Image size (um) [1]	88.8 x 88.8 x 328.4	91.3 x 90.8 x 268.1	90.3 x 90.8 x 207.8	90.3 x 90.3 x 267.7
Cortical depth of upper end (um)	1710	1620	1570	1420
Number of model nodes	9612	22105	14641	21144
Number of constituents	48	73	63	86
Pyramidal neurons	3	4	1	2
Interneurons	0	0	0	0
Non-typed neurons	0	1	0	0
Orphan neurites	45	68	62	84
Gliaform cells	0	0	0	0
Blood capillaries	0	0	0	0
Total length (um) [2]	3113.3	6727.3	4393.0	7071.2
Pyramidal process (um)	1318.0	3695.8	1166.6	2819.6
Interneuron process (um)	0.0	0.0	0.0	0.0
Non-typed neuron process (um)	0.0	470.1	0.0	0.0
Orphan neurite (um)	1795.3	2561.4	3226.4	4251.6
Gliaform cell process (um)	0.0	0.0	0.0	0.0
Blood capillary (um)	0.0	0.0	0.0	0.0
Number of neurite segments	94	189	115	183
Neurite curvature (um^{-1}) [3]	0.64 (0.33)	0.62 (0.34)	0.54 (0.20)	0.53 (0.25)
Neurite radius (um) [3]	0.54 (0.62)	0.46 (0.46)	0.41 (0.18)	0.41 (0.20)
Number of spines	746	1887	1229	1656
Spine curvature (um^{-1}) [3]	1.44 (0.61)	1.43 (0.61)	1.38 (0.60)	1.46 (0.62)
Spine radius (um) [3]	0.20 (0.05)	0.20 (0.06)	0.22 (0.06)	0.20 (0.06)
Spine length (um) [3]	1.10 (0.77)	1.16 (0.73)	1.34 (0.78)	1.20 (0.77)
Spine density (um^{-1}) [4]	0.268	0.314	0.302	0.253

[1] Image width x height x number of slices

[2] Spine length is not included.

[3] Mean (standard deviation)

[4] Spine density = number of spines / total length of spiny dendrite

Supplementary Table 3. Statistics of datasets and Cartesian coordinate models. (**G**) Control case N3.

Dataset name	N3A	N3B	N3C	N3D	N3E
Beamtime start date	2018.10.5	2018.10.5	2018.10.5	2018.10.5	2018.10.5
Image size (pixel) [1]	1840 x 1860 x 11660	1850 x 1860 x 6735	1930 x 2120 x 7967	1860 x 1870 x 5500	1860 x 1860 x 6727
Image size (um) [1]	89.8 x 90.8 x 569.0	90.3 x 90.8 x 328.7	94.2 x 103.5 x 388.8	90.8 x 91.3 x 268.4	90.8 x 90.8 x 328.3
Cortical depth of upper end (um)	2810	3060	3090	3240	3280
Number of model nodes	5085	9862	7228	4712	10533
Number of constituents	20	43	41	32	64
Pyramidal neurons	1	2	2	4	1
Interneurons	0	0	1	0	0
Non-typed neurons	0	0	0	0	0
Orphan neurites	19	40	37	28	63
Gliaform cells	0	0	0	0	0
Blood capillaries	0	1	1	0	0
Total length (um) [2]	2315.6	4362.5	3470.0	2519.1	4505.8
Pyramidal process (um)	1436.3	2029.4	2104.2	1529.4	1680.5
Interneuron process (um)	0.0	0.0	7.1	0.0	0.0
Non-typed neuron process (um)	0.0	0.0	0.0	0.0	0.0
Orphan neurite (um)	879.3	2242.3	1276.8	989.7	2825.3
Gliaform cell process (um)	0.0	0.0	0.0	0.0	0.0
Blood capillary (um)	0.0	90.8	82.0	0.0	0.0
Number of neurite segments	59	132	112	90	125
Neurite curvature (um^{-1}) [3]	0.48 (0.22)	0.44 (0.22)	0.47 (0.18)	0.42 (0.21)	0.49 (0.26)
Neurite radius (um) [3]	0.48 (0.33)	0.52 (0.37)	0.44 (0.30)	0.58 (0.56)	0.39 (0.20)
Number of spines	289	767	409	230	728
Spine curvature (um^{-1}) [3]	1.43 (0.67)	1.38 (0.69)	1.35 (0.71)	1.37 (0.61)	1.30 (0.64)
Spine radius (um) [3]	0.22 (0.07)	0.22 (0.06)	0.22 (0.06)	0.20 (0.06)	0.21 (0.06)
Spine length (um) [3]	0.91 (0.66)	0.85 (0.60)	0.71 (0.49)	0.75 (0.57)	0.82 (0.59)
Spine density (um^{-1}) [4]	0.138	0.195	0.151	0.123	0.192

[1] Image width x height x number of slices

[2] Spine length is not included.

[3] Mean (standard deviation)

[4] Spine density = number of spines / total length of spiny dendrite

Supplementary Table 3. Statistics of datasets and Cartesian coordinate models. (**H**) Control case N4.

Dataset name	N4A	N4B	N4C
Beamtime start date	2018.10.5	2018.10.5	2018.10.5
Image size (pixel) [1]	1860 x 1850 x 5496	1850 x 1850 x 6731	1840 x 1870 x 5492
Image size (um) [1]	90.8 x 90.3 x 268.2	90.3 x 90.3 x 328.5	89.8 x 91.3 x 268.0
Cortical depth of upper end (um)	1970	1870	1660
Number of model nodes	10783	7599	7129
Number of constituents	84	63	81
Pyramidal neurons	4	2	2
Interneurons	0	0	1
Non-typed neurons	0	0	2
Orphan neurites	80	61	76
Gliaform cells	0	0	0
Blood capillaries	0	0	0
Total length (um) [2]	6915.4	5088.1	4658.1
Pyramidal process (um)	3971.6	1195.3	506.3
Interneuron process (um)	0.0	0.0	225.1
Non-typed neuron process (um)	0.0	0.0	210.6
Orphan neurite (um)	2943.8	3892.8	3716.1
Gliaform cell process (um)	0.0	0.0	0.0
Blood capillary (um)	0.0	0.0	0.0
Number of neurite segments	220	151	152
Neurite curvature (um^{-1}) [3]	0.35 (0.18)	0.32 (0.19)	0.36 (0.19)
Neurite radius (um) [3]	0.65 (0.82)	0.65 (0.56)	0.53 (0.34)
Number of spines	418	344	115
Spine curvature (um^{-1}) [3]	1.19 (0.61)	1.07 (0.57)	1.07 (0.57)
Spine radius (um) [3]	0.24 (0.07)	0.24 (0.07)	0.24 (0.07)
Spine length (um) [3]	0.59 (0.42)	0.63 (0.44)	0.69 (0.48)
Spine density (um^{-1}) [4]	0.080	0.091	0.055

[1] Image width x height x number of slices

[2] Spine length is not included.

[3] Mean (standard deviation)

[4] Spine density = number of spines / total length of spiny dendrite
